# Evolutionary relationships and population genetics of the Afrotropical leaf-nosed bats (Chiroptera, Hipposideridae)

**DOI:** 10.3897/zookeys.929.50240

**Published:** 2020-04-22

**Authors:** Bruce D. Patterson, Paul W. Webala, Tyrone H. Lavery, Bernard R. Agwanda, Steven M. Goodman, Julian C. Kerbis Peterhans, Terrence C. Demos

**Affiliations:** 1 Negaunee Integrative Research Center, Field Museum of Natural History, Chicago IL 60605, USA Field Museum of Natural History Chicago United States of America; 2 Department of Forestry and Wildlife Management, Maasai Mara University, Narok, Kenya Maasai Mara University Narok Kenya; 3 Threatened Species Recovery Hub, Fenner School of Environment and Society, The Australian National University, Canberra, ACT, Australia The Australian National University Canberra Australia; 4 Mammalogy Section, National Museums of Kenya, Nairobi, Kenya National Museums of Kenya Nairobi Kenya; 5 Association Vahatra, BP 3972, Antananarivo 101, Madagascar National Museums of Kenya Antananarivo Madagascar; 6 College of Arts and Sciences, Roosevelt University, Chicago, IL, 60605, USA Roosevelt University Chicago United States of America

**Keywords:** cryptic species, mtDNA, nuclear introns, Paleotropical, phylogeny, species delimitation, systematics

## Abstract

The Old World leaf-nosed bats (Hipposideridae) are aerial and gleaning insectivores that occur throughout the Paleotropics. Both their taxonomic and phylogenetic histories are confused. Until recently, the family included genera now allocated to the Rhinonycteridae and was recognized as a subfamily of Rhinolophidae. Evidence that Hipposideridae diverged from both Rhinolophidae and Rhinonycteridae in the Eocene confirmed their family rank, but their intrafamilial relationships remain poorly resolved. We examined genetic variation in the Afrotropical hipposiderids *Doryrhina*, *Hipposideros*, and *Macronycteris* using relatively dense taxon-sampling throughout East Africa and neighboring regions. Variation in both mitochondrial (cyt-b) and four nuclear intron sequences (ACOX2, COPS, ROGDI, STAT5) were analyzed using both maximum likelihood and Bayesian inference methods. We used intron sequences and the lineage delimitation method BPP—a multilocus, multi-species coalescent approach—on supported mitochondrial clades to identify those acting as independent evolutionary lineages. The program StarBEAST was used on the intron sequences to produce a species tree of the sampled Afrotropical hipposiderids. All genetic analyses strongly support generic monophyly, with *Doryrhina* and *Macronycteris* as Afrotropical sister genera distinct from a Paleotropical *Hipposideros*; mitochondrial analyses interpose the genera *Aselliscus*, *Coelops*, and *Asellia* between these clades. Mitochondrial analyses also suggest at least two separate colonizations of Africa by Asian groups of *Hipposideros*, but the actual number and direction of faunal interchanges will hinge on placement of the unsampled African-Arabian species *H. megalotis*. Mitochondrial sequences further identify a large number of geographically structured clades within species of all three genera. However, in sharp contrast to this pattern, the four nuclear introns fail to distinguish many of these groups and their geographic structuring disappears. Various distinctive mitochondrial clades are consolidated in the intron-based gene trees and delimitation analyses, calling into question their evolutionary independence or else indicating their very recent divergence. At the same time, there is now compelling genetic evidence in both mitochondrial and nuclear sequences for several additional unnamed species among the Afrotropical *Hipposideros*. Conflicting appraisals of differentiation among the Afrotropical hipposiderids based on mitochondrial and nuclear loci must be adjudicated by large-scale integrative analyses of echolocation calls, quantitative morphology, and geometric morphometrics. Integrative analyses will also help to resolve the challenging taxonomic issues posed by the diversification of the many lineages associated with *H. caffer* and *H. ruber*.

## Introduction

The Old World leaf-nosed bats, family Hipposideridae, currently include seven genera and 90 species of insectivorous bats distributed over much of the Paleotropics (Monadjem 2019; Simmons and Cirranello 2019). Both the taxonomic and phylogenetic histories of this family are confused. Throughout much of its history (e.g., Koopman 1989), Hipposideridae was considered either a subfamily of the Rhinolophidae (the horseshoe bats) or as its sister family within the Rhinolophoidea. Recently, however, the “trident bats” (*Cloeotis*, *Paratriaenops*, *Rhinonicteris*, and *Triaenops*) were shown to comprise a family-ranked group, the Rhinonycteridae, which is separate from and sister to the Hipposideridae (Foley et al. 2015; Armstrong et al. 2016). Even the genus *Hipposideros* Gray, 1831, as it was traditionally understood, appears paraphyletic with respect to the allied genera *Asellia*, *Aselliscus*, *Coelops*, and *Anthops* (Foley et al. 2015; Amador et al. 2018). Re-validation of *Macronycteris* Gray, 1866 and *Doryrhina* Peters, 1871 for groups of Afrotropical endemic species more closely related to each other than to African and Asian members of *Hipposideros* sensu stricto resolved a number of those issues (Foley et al. 2017).

**Figure 1.**
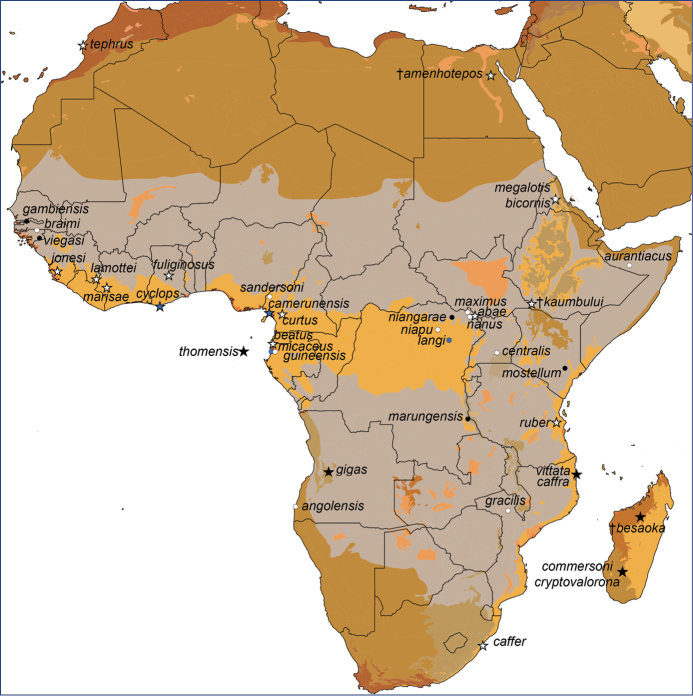
Type localities for Afrotropical hipposiderids: *Doryrhina*, blue symbols; *Hipposideros*, white symbols; *Macronycteris*, black symbols. Stars denote valid species, whereas circles indicate taxa considered as subspecies or synonyms. Localities are projected onto the biome map of Olson et al. (2001). Taxa depicted are: *Hipposideros abae* J. A. Allen,1917; †Hipposideros (Pseudorhinolophus) amenhotepos Gunnell, Winkler, Miller, Head, El-Barkooky, Gawad, Sanders & Gingerich, 2015; *Phyllorhina angolensis* Seabra, 1898; Hipposideros caffer var. aurantiaca De Beaux, 1924; *Hipposideros beatus* K. Andersen, 1906; †*Hipposideros besaoka* Samonds, 2007; *Phyllorrhina bicornis* Heuglin, 1861; *Hipposideros braima* Monard, 1939; *Hipposideros caffer* Sundevall, 1846; *Phyllorhina caffra* Peters, 1852; *Hipposideros camerunensis* Eisentraut, 1956; *Hipposideros caffer centralis* K. Andersen, 1906; *Rhinolophus Commersonii* É. Geoffroy, 1813; *Hipposideros cryptovalorona* Goodman, Schoeman, Rakotoarivelo & Willows-Munro, 2016; *Hipposideros curtus* G. M. Allen, 1921; *Phyllorrhina cyclops* Temminck, 1853; *Phyllorrhina fuliginosa* Temminck, 1853; *Hipposideros gigas gambiensis* K. Andersen, 1906; *Rhinolophus gigas* Wagner, 1845; *Phyllorrhina gracilis* Peters, 1852; *Hipposideros caffer guineensis* K. Andersen, 1906; *Hipposideros jonesi* Hayman, 1947; †*Hipposideros kaumbului* Wesselman, 1984; *Hipposideros lamottei* Brosset, 1985; *Hipposideros langi* J. A. Allen, 1917; *Hipposideros marisae* Aellen, 1954; *Phyllorhina Commersoni*, var. marungensis Noack, 1887; *Hipposideros beatus maximus* Verschuren, 1957; *Phyllorrhina megalotis* Heuglin, 1861; *Rhinolophus micaceus* de Winton, 1897; *HipposiderosCommersoni mostellum* Thomas, 1904; *Hipposideros nanus* J. A. Allen, 1917; *Hipposideros gigas niangarae* J. A. Allen, 1917; *Hipposideros caffer niapu* J. A. Allen, 1917; *Phyllorrhina rubra* Noack, 1893; *Hipposideros sandersoni* Sanderson, 1937; *Hipposideros tephrus* Cabrera, 1906; *Phyllorhina Commersoni*, var. thomensis Bocage, 1891; *Hipposideros gigas viegasi* Monard, 1939; *Phyllorhina vittata* Peters, 1852.

The species richness of *Doryrhina*, *Macronycteris*, and *Hipposideros* differs widely. Most authors recognize two species of *Doryrhina* (*D. cyclops* and *D. camerunensis*), five species of *Macronycteris* (*M. commersoni*, *M. cryptovalorona*, *M. gigas*, *M. thomensis*, and *M. vittata*), and 83 species of *Hipposideros*, 10 of which occur in Africa (Monadjem 2019; Simmons and Cirranello 2019). These are *H. beatus*, *H. caffer*, *H. curtus*, *H. fuliginosus*, *H. lamottei*, *H. ruber*, and *H. tephrus* in the *bicolor* group of *Hipposideros*; *H. jonesi* and *H. marisae* in the *speoris* group, and *H. megalotis* in the *megalotis* group (Hill 1963; Murray et al. 2012; Monadjem 2019). In addition, three extinct species of hipposiderid are known from the region: †*Macronycteris besaoka* (Madagascar), †*Hipposideros amenhotepos* (Egypt), and †*H. kaumbului* (Ethiopia). Type localities for valid species, subspecies, and synonyms for these three genera in Africa and Madagascar appear in Figure 1; after the removal of *Doryrhina* and *Macronycteris* taxa, group assignments for the species remaining in *Hipposideros* appear in Table 1.

**Table 1.** Species groups of *Hipposideros* (modified from Murray et al. 2012 to include newly recognized forms and to remove species now recognized in *Doryrhina* and *Macronycteris*).

**Armiger group**	calcaratus subgroup	*H. macrobullatus*	*H. lankadiva*
*H. alongensis*	*H. calcaratus* c	*H. maggietaylorae*	*H. lekaguli*
*H. armiger*	*H. cervinus* c	*H. nequam*	*H. pelingensis*
*H. griffini* a	*H. coxi* c	*H. obscurus*	**Larvatus group**
*H. pendelburyi* a	*H. galeritus* c	*H. orbiculus*	*H. grandis*
*H. turpis*	ruber subgroup	*H. papua*	“*H. khasiana*” a,g
**Bicolor group**	*H. abae* d	*H. pygmaeus*	*H. larvatus*
ater subgroup	*H. beatus* e	**Boeadii group**	*H. madurae*
*H. ater* b	*H. caffer* e	*H. boeadii*	*H. sorenseni*
*H. atrox* a	*H. fuliginosus* e	**Cyclops group** f	*H. sumbae*
*H. bicolor* b	*H. lamottei* e	*H. corynophyllus*	**Megalotis group**
*H. breviceps* b	*H. ruber* e	*H. edwardshilli*	*H. megalotis*
*H. cineraceus* b	*H. tephrus* a,e	*H. muscinus*	**Pratti group**
*H. coronatus* b	subgroup uncertain	*H. semoni*	*H. lylei*
*H. dyacorum* b	*H. cruminiferus*	*H. stenotis*	*H. pratti*
*H. einnaythu* a,b	*H. curtus*	*H. wollastoni*	*H. scutinares*
*H. halophyllus* b	*H. doriae*	**Diadema group**	**Speoris group**
*H. khaokhouayensis* b	*H. durgadasi*	*H. demissus*	*H. jonesi* h
*H. nicobarulae* a,b	*H. fulvus*	*H. diadema*	*H. marisae* g
*H. pomona* b	*H. gentilis* a	*H. dinops*	*H. speoris*
*H. ridleyi* b	*H. hypophyllus*	*H. inexpectatus*	
*H. rotalis* b	*H. kunzi* a	*H. inornatus*	

(Endnotes) a Added to species list subsequent to Murray et al. (2012) b Recognized in the Ater species group by Monadjem (2019) c Recognized in the Calcaratus species group by Monadjem (2019) d Formerly listed in the Speoris group but transferred to the Ruber group by Monadjem (2019) e Recognized in the Ruber species group by Monadjem (2019) f *H. cyclops* and *H. camerunensis* are now recognized as members of *Doryrhina*; listed species were treated as *Doryrhina in*Monadjem (2019) on the basis of similar morphology but were recognized as the Muscinus group by Tate (1941); they might represent an unnamed genus or subgenus. g Invalid name accorded to what is likely a real biological entity (cf. Monadjem 2019) h Formerly in the Bicolor species group but transferred to the Speoris group by Monadjem (2019).

As suggested by their checkered taxonomic history, phylogenetic understanding of the Hipposideridae has slowly come into focus. *Doryrhina* and *Macronycteris* are two of a dozen generic-group names that were synonymized with *Hipposideros* for all of the 20^th^ century (Miller 1907; Allen 1939; Koopman 1994). Instead of subgenera, taxonomists used the species groups delineated by Andersen (1918) and refined by Tate (1941) and Hill (1963) in their generic revisions based on morphology. Assessment of rhinolophoid relationships using an intron supermatrix (Eick et al. 2005) confirmed the early divergence of hipposiderids and rhinolophids (estimated at 41 Ma), thereby substantiating their rank as a separate families. Despite earlier suppositions that the area of origin for Hipposideridae was in Asia (Koopman 1970; Bogdanowicz and Owen 1998) or Australia (Hand and Kirsch 1998), Eick et al. (2005) clearly demonstrated the ancestry of the family (and superfamily) was in Africa. A recent supermatrix analysis with the most comprehensive taxonomic sampling (42 species; Amador et al. 2018) confirmed the early divergence of hipposiderids and rhinolophids at 41.3 Ma, but this analysis questioned the validity of both *Doryrhina* and *Macronycteris*. Amador et al. attributed the paraphyly of *Hipposideros* sensu lato documented by Foley et al. (2015) to their limited taxonomic sampling. Amador et al. (2018) also challenged the integrity of the *commersoni*, *cyclops*, *speoris*, and *bicolor* species groups, arguing that all African species save for *H. jonesi* belonged in a single, exclusively African species group.

Although new species of hipposiderids are regularly discovered and described in Asia (Robinson et al. 2003; Guillen-Servent and Francis 2006; Bates et al. 2007; Douangboubpha et al. 2011; Thong et al. 2012; Murray et al. 2018), the pace of discovery has been much slower in Africa. Only one extant species has been described since the recognition of *Hipposideros lamottei* (Brosset 1985 [“1984”]), and that one was from Madagascar (Goodman et al. 2016). Surveys of mitochondrial sequences from African hipposiderids have strongly suggested that supposedly widespread species such as *Hipposideros caffer* and *H. ruber* actually represent complexes of cryptic species (Vallo et al. 2008, 2011; Monadjem et al. 2013). Phylogenetic analyses (e.g., Vallo et al. 2008) show that these named species complexes are not monophyletic, resolving clades comprised of bats identified as both *H. caffer* and *H. ruber*. These studies have characterized the clades in both morphological and genetic terms, even establishing them in sympatry (see also Vallo et al. 2011). However, the uncertain relationship of the identified clades to the many names already proposed for Afrotropical hipposiderids, many based on incomplete or formalin-preserved specimens, has precluded formally naming them. Incomplete geographic sampling and the lack of evidence from nuclear genes for these populations has also clouded interpretations of this mitochondrial diversity.

Our field surveys in Eastern Africa and adjoining regions offer a new basis for considering the taxonomy and phylogenetics of Afrotropical hipposiderids. We sought to answer these questions: (1) Is there compelling evidence to support the recognition of *Doryrhina* and *Macronycteris* as distinct Afrotropical genera alongside the Paleotropical *Hipposideros*? (2) Which species belong to these groups? (3) Are the traditional species groups of African hipposiderids monophyletic? Using both mitochondrial and nuclear intron sequences, we also evaluate the question of cryptic species among African hipposiderids and the possibility of mitochondrial-nuclear discordance.

## Material and methods

### Selection of taxa and sampling

Our genetic dataset is based on 453 hipposiderid individuals, the vast majority being represented by museum vouchers. We generated original genetic data from 319 individuals collected at 102 georeferenced localities, and complemented them with 134 mitochondrial sequences from 90 localities downloaded from GenBank (we obtained new sequence data for five individuals with prior GenBank records; see Suppl. material 1: Figure S1 and Appendix I). All individuals were sequenced for Cytochrome-b (cyt-b) in order to maximize assessment of genetic diversity; however, redundant haplotypes were removed for subsequent phylogenetic analyses (see Appendix I for complete list of individuals sequenced). The bats newly sequenced for this study were obtained over several decades in the course of small mammal surveys across sub-Saharan Africa and Madagascar, with relatively dense sampling in East Africa. Initial assignment of East African individuals to species was determined using meristic, mensural, and qualitative characters published in the bat keys of Thorn et al. (2009) and Patterson and Webala (2012). Collection methods followed mammal guidelines for the use of wild mammals in research and education (Sikes and the Animal Care and Use Committee of the American Society of Mammalogists 2016) and the most recent collections were approved under Field Museum of Natural History’s IACUC #2012-003. Only GenBank records for cyt-b were available for records of the Arabian-North African hipposiderid *Asellia*, which was included for context in the phylogenetic analyses. Lacking information from nuclear introns, we draw no firm conclusions from their placement and do not discuss *Asellia* in this paper (see Benda et al. 2011; Bray and Benda 2016).

Appendix I contains the institutions and voucher numbers, GenBank accession numbers, and locality information for our samples. The fact that museum voucher specimens were used wherever possible for the genetic analyses permits the genetic analysis to serve as a foundation for integrative taxonomic analyses of dental, cranial, and skeletal variation, using the same specimens. To avoid adding to current taxonomic confusion, we take a conservative approach in assigning names to clades in our analyses. Where a clade’s taxonomic identity was ambiguous or unknown, we referred to it simply as a numbered clade. Integrative taxonomic diagnoses of the various clades supported by our analyses will be necessary to determine which, if any, existing names may apply to them. However, to relate our results to those of earlier studies of African *Hipposideros* (Vallo et al. 2008, 2011; Monadjem et al. 2013), we cross-referenced specimens used in two or more analyses to equate the various non-binomial names that have been applied to these cryptic lineages.

### DNA extraction, amplification, and sequencing

Genomic DNA from preserved tissue samples was extracted using the Wizard SV 96 Genomic DNA Purification System (Promega Corporation, WI, USA). Fresh specimens were sequenced for mitochondrial cytochrome-b (cyt-b), using the primer pair LGL 765F and LGL 766R (Bickham et al. 1995; Bickham et al. 2004), and four unlinked autosomal nuclear introns: ACOX2 intron 3, COPS7A intron 4, ROGDI intron 7 (Salicini et al. 2011), and STAT5B (Matthee et al. 2001) for hipposiderid specimens and the sister group *Triaenops afer* (Rhinonycteridae; see Table 1 for primer information). PCR amplification, thermocycler conditions, and sequencing were identical to Patterson et al. (2018) and Demos et al. (2018). Sequences were assembled and edited using GENEIOUS PRO v.11.1.5 (Biomatters Ltd). Sequence alignments were made using MUSCLE (Edgar 2004) with default settings in GENEIOUS. Protein coding data from cyt-b were translated to amino acids to determine codon positions and confirm the absence of premature stop codons, deletions, and insertions. Several gaps were incorporated in the nuclear intron alignments, but their positions were unambiguous.

Sequence alignments used in this study have been deposited on the FIGSHARE data repository (https://doi.org/10.6084/m9.figshare.11936250). All newly generated sequences were deposited in GenBank with accession numbers MT149315–MT149893 (see also Appendix I).

### Phylogenetic analyses

jMODELTEST2 (Darriba et al. 2012) on CIPRES Science Gateway v. 3.3 (Miller et al. 2010) was used to determine the sequence substitution models that best fit the data using the Bayesian Information Criterion (BIC) for cyt-b and the four nuclear introns. PARTITIONFINDER2 (Lanfear et al. 2016) on CIPRES was used to determine the sequence substitution models for the concatenated alignment of four nuclear introns using the Bayesian Information Criterion (BIC) with the ‘greedy’ search algorithm. Uncorrected sequence divergences (*p*-distances) between and within species/clades were calculated for cyt-b using MEGA X v. 10.0.5 (Kumar et al. 2018). Maximum-likelihood (ML) analyses were performed using the program IQ-TREE v. 1.6.10 (Chernomor et al. 2016; Nguyen et al. 2015) on the CIPRES portal for separate gene trees (cyt-b, ACOX2, COPS7A, ROGDI, and STAT5B) and a concatenated alignment, partitioned by gene, using the four nuclear introns. As in Hillis and Bull (1993), nodes supported by bootstrap values (BP) ≥ 70% were considered strongly supported. Gene tree analyses under a Bayesian Inference (BI) framework were inferred in MRBAYES v. 3.2.7 (Ronquist et al. 2012) on the CIPRES portal for the same set of genes as the ML analyses. Two independent runs were conducted in MrBayes, and nucleotide substitution models were unlinked across partitions for each nuclear locus in the concatenated alignment. Four Markov chains were run for 1 × 10^8^ generations for individual gene trees, and 2 × 10^7^ generations for the concatenated analysis, using default heating values and sampled every 1000^th^ generation. A conservative 25% burn-in was applied and stationarity of the MRBAYES results was assessed in Tracer v. 1.7 (Rambaut et al. 2018). Majority-rule consensus trees were constructed for each Bayesian analysis. Following Erixon et al. (2003), nodes supported by posterior probabilities (PP) ≥0.95 were considered strongly supported.

Haplotype networks for cyt-b were inferred using the median-joining network algorithm in PopArt v. 1.7 (Leigh and Bryant 2015). Separate analyses were carried out for the following clades, each consisting of four subclades: (1) *Doryhina* (*D. camerunensis*, D. cf. camerunensis, *D. cyclops*1, and *D. cyclops*2); (2) *Macronycteris* (*M. commersoni*, *M. cryptovalorona*, *M. gigas*, and *M. vittata*); (3) *Hipposideros caffer*1–4; (4) *Hipposideros caffer*5–8; and (5) *Hipposideros ruber*1–4.

Hipposiderid taxa included in the species tree analyses were assigned to either species or numbered clades based on clade support in the ML and BI gene-tree analyses of the cyt-b dataset. This in turn identified populations to be used as ‘candidate species’ in a coalescent-based species-tree approach implemented in StarBEAST2 (Ogilvie et al. 2017), an extension of BEAST v. 2.5.1 (Drummond et al. 2012; Bouckaert et al. 2014). Species tree analysis was conducted using the four nuclear intron alignments. Substitution, clock, and tree models were unlinked across all loci. A lognormal relaxed-clock model was applied to each locus under a Yule tree prior and a linear with constant root population size model. Four independent replicates were run with random starting seeds, and chain lengths of 1 × 10^8^ generations and parameters were sampled every 5,000 steps. For the StarBEAST2 analyses, evidence of convergence and stationarity of posterior distributions of model parameters was assessed based on ESS values >200 and examination of trace files in Tracer v. 1.7. The burn-in was set at 10% and separate runs were assembled using LOGCOMBINER v. 2.5.1 and TREEANNOTATOR v. 2.5.1 (Rambaut et al. 2018).

### Coalescent lineage delimitation

Based on the well supported clades obtained in the cyt-b gene tree analyses and available intron samples, a lineage delimitation scenario with 18 candidate species was tested. We inferred the evolutionary isolation of their gene pools using the phased nuclear DNA dataset (ACOX2, COPS7A, ROGDI, and STAT5A; 104 individuals) for joint independent lineage delimitation and species-tree estimation evaluated under the multi-species coalescent model using the program BPP v. 3.3 (Yang and Rannala 2014; Rannala and Yang 2017). This analysis was carried out to guide future investigations of the species status of evolutionarily isolated lineages inferred here. Supported lineages will be examined using an integrative species taxonomic approach, including morphological, morphometric, and acoustic characters, as well as ectoparasite associations and distributional data. Species/clade memberships for BPP were identical to individuals assigned to lineages in the species tree analyses. The validity of our assignment of individuals to populations was tested using the guide-tree-free algorithm (A11) in BPP. Because the probability of delimitation by BPP is sensitive to selected parameters (Leaché and Fujita 2010; Yang 2015), we evaluated two independent runs for each of four different combinations of divergence depth and effective population sizes priors (*τ* and *θ*, respectively; Table 2). Two independent MCMC chains were run for 5 × 10^4^ generations. The burn-in was 20% and samples drawn every 50^th^ generation. In total, eight BPP runs were carried out using four phased nuclear intron alignments. Lineages were considered to be statistically well supported when the delimitation posterior probabilities generated were ≥0.95 under all four combinations of priors.

**Table 2.** Primer information and chosen substitution models for regions amplified in this study. Substitution models before “/” are the best-supported models inferred by jMODELTEST2 and models after “/” indicate those inferred by PARTITIONFINDER2 for the concatenated intron alignment.

Primer name	Sequence	Primer publication	Substitution model
ACOX2-3-F	5’-CCTSGGCTCDGAGGAGCAGAT-3’	Salicini et al. 2011	K80+G / K81+G
ACOX2-3-R	5’-GGGCTGTGHAYCACAAACTCCT-3’		
COPS7A-4-F	5’-TACAGCATYGGRCGRGACATCCA-3’	Salicini et al. 2011	HKY / K80
COPS7A-4-R	5’-TCACYTGCTCCTCRATGCCKGACA-3’		
ROGDI-7-F	5’-CTGATGGAYGCYGTGATGCTGCA-3’	Salicini et al. 2011	K80+G / K81+G
ROGDI-7-R	5’-CACGGTGAGGCASAGCTTGTTGA-3’		
STAT5B-16-F	5’--CTGCTCATCAACAAGCCCGA-3’	Matthee et al. 2001	GTR+G / K81+G
STAT5B-16-R	5’-GGCTTCAGGTTCCACAGGTTGC-3’		
cyt-b-LGL-765-F	5’-GGCTTCAGGTTCCACAGGTTGC-3’	Trujillo et al. 2009	GTR+I+G
cyt-b -LGL-766-R	5’-GTTTAATTAGAATYTYAGCTTTGGG-3’		

**Table 3.** Prior Schemes (PS) used in BPP analyses. Prior distributions on τ represent two relative divergence depths (deep and shallow) and on θ represent two relative effective population sizes (large and small) scaling mutation rates.

PS	Effective pop. size	Divergence depth	Gamma distribution for prior
1	Large	Deep	*θ* = Γ [1, 10] and *τ* = Γ [1, 10]
2	Large	Shallow	*θ* = Γ [1, 10] and *τ* = Γ [2, 2000]
3	Small	Shallow	*θ* = Γ [2, 2000] and *τ* = Γ [2, 2000]
4	Small	Deep	*θ* = Γ [2, 2000] and *τ* = Γ [1, 10]

## Results

In terms of cyt-b sequence divergence, clades within *Doryrhina* are separated by 3.0–5.7% genetic distances, whereas less than 3% separates the four recognized species of *Macronycteris*. Between Afrotropical *Hipposideros*, the greatest distances separate *H. jonesi* from other lineages (13.4–16.1%). The various numbered clades allied to *Hipposideros caffer* differ from one another in cyt-b sequences by 2.5–10.3% and clades allied to *H. ruber* differ by 3.0–8.2% (Table 4).

**Table 4.** Uncorrected cyt-b p-distances between (off diagonal) and within (on diagonal) Afrotropical hipposiderid clades, showing the number of base differences per site averaged over all sequence pairs between groups. The analysis involved 386 nucleotide sequences and all ambiguous positions were removed; na (not available) reflects a sample size of one individual.

		[1]	[2]	[3]	[4]	[5]	[6]	[7]	[8]	[9]	[10]	[11]	[12]	[13]	[14]	[15]
[1]	*Doryrhina camerunensis*	**0.003**														
[2]	Doryrhina cf. camerunensis	0.055	**na**													
[3]	*Doryrhina cyclops* 1	0.057	0.048	**0.008**												
[4]	*Doryrhina cyclops* 2	0.055	0.041	0.030	**0.006**											
[5]	*Hipposideros abae*	0.176	0.173	0.168	0.166	**0.033**										
[6]	*Hipposideros beatus* 1	0.152	0.156	0.160	0.152	0.116	**0.007**									
[7]	*Hipposideros beatus* 2	0.146	0.149	0.151	0.147	0.117	0.044	**0.006**								
[8]	*Hipposideros caffer* 1	0.157	0.154	0.150	0.148	0.108	0.103	0.106	**0.006**							
[9]	*Hipposideros caffer* 2	0.153	0.150	0.150	0.147	0.106	0.096	0.108	0.045	**0.01**						
[10]	*Hipposideros caffer* 3	0.152	0.148	0.150	0.149	0.108	0.110	0.111	0.046	0.052	**0.011**					
[11]	*Hipposideros caffer* 4	0.162	0.159	0.160	0.154	0.106	0.105	0.114	0.077	0.078	0.079	**0.018**				
[12]	*Hipposideros caffer* 5	0.150	0.155	0.159	0.148	0.113	0.091	0.096	0.095	0.101	0.103	0.098	**0.005**			
[13]	*Hipposideros caffer* 6	0.155	0.156	0.164	0.153	0.112	0.093	0.102	0.090	0.097	0.099	0.094	0.028	**0.011**		
[14]	*Hipposideros caffer* 7	0.151	0.154	0.161	0.148	0.108	0.084	0.094	0.094	0.094	0.098	0.096	0.025	0.032	**0.011**	
[15]	*Hipposideros caffer* 8	0.154	0.155	0.160	0.152	0.111	0.090	0.092	0.092	0.095	0.102	0.093	0.033	0.039	0.029	**0.021**
[16]	*Hipposideros fuliginosus*	0.155	0.149	0.154	0.142	0.101	0.095	0.096	0.078	0.084	0.087	0.094	0.088	0.086	0.080	0.085
[17]	*Hipposideros jonesi*	0.153	0.143	0.154	0.147	0.160	0.145	0.138	0.135	0.140	0.140	0.139	0.134	0.134	0.137	0.139
[18]	*Hipposideros lamottei*	0.171	0.173	0.174	0.163	0.106	0.097	0.118	0.094	0.086	0.095	0.097	0.057	0.059	0.052	0.060
[19]	Hipposideros cf. lamottei	0.158	0.158	0.156	0.149	0.107	0.103	0.107	0.091	0.099	0.095	0.097	0.053	0.058	0.052	0.054
[20]	*Hipposideros marisae*	0.177	0.180	0.178	0.172	0.159	0.153	0.153	0.144	0.148	0.140	0.152	0.148	0.144	0.154	0.159
[21]	*Hipposideros ruber* 1	0.155	0.151	0.152	0.142	0.104	0.099	0.101	0.081	0.084	0.085	0.086	0.090	0.081	0.082	0.082
[22]	*Hipposideros ruber* 2	0.155	0.155	0.156	0.142	0.103	0.097	0.102	0.081	0.082	0.089	0.089	0.086	0.078	0.083	0.081
[23]	*Hipposideros ruber* 3	0.159	0.149	0.155	0.147	0.105	0.094	0.101	0.084	0.082	0.096	0.088	0.090	0.083	0.082	0.082
[24]	*Hipposideros ruber* 4	0.153	0.147	0.151	0.141	0.100	0.094	0.097	0.073	0.073	0.085	0.082	0.083	0.077	0.078	0.078
[25]	Hipposideros cf. ruber	0.164	0.161	0.161	0.153	0.099	0.094	0.100	0.086	0.084	0.095	0.093	0.081	0.080	0.080	0.083
[26]	*Macronycteris commersoni*	0.157	0.156	0.155	0.145	0.164	0.159	0.154	0.164	0.169	0.168	0.171	0.166	0.170	0.167	0.162
[27]	*Macronycteris cryptovalorona*	0.147	0.143	0.147	0.142	0.157	0.152	0.146	0.156	0.162	0.161	0.165	0.159	0.164	0.158	0.157
[28]	*Macronycteris gigas*	0.151	0.149	0.154	0.144	0.164	0.163	0.158	0.162	0.168	0.165	0.170	0.165	0.171	0.164	0.165
[29]	*Macronycteris vittata*	0.147	0.148	0.149	0.140	0.162	0.158	0.147	0.160	0.161	0.166	0.165	0.164	0.169	0.164	0.163

**Table 4.** Continued.

		[16]	[17]	[18]	[19]	[20]	[21]	[22]	[23]	[24]	[25]	[26]	[27]	[28]	[29]
[16]	*Hipposideros fuliginosus*	**0.003**													
[17]	*Hipposideros jonesi*	0.137	**0.008**												
[18]	*Hipposideros lamottei*	0.100	0.161	**0.038**											
[19]	Hipposideros cf. lamottei	0.091	0.142	0.054	**0.006**										
[20]	*Hipposideros marisae*	0.145	0.092	0.156	0.158	**na**									
[21]	*Hipposideros ruber* 1	0.082	0.144	0.093	0.087	0.152	**0.013**								
[22]	*Hipposideros ruber* 2	0.084	0.147	0.093	0.091	0.157	0.030	**0.007**							
[23]	*Hipposideros ruber* 3	0.082	0.142	0.085	0.088	0.158	0.052	0.057	**0.022**						
[24]	*Hipposideros ruber* 4	0.078	0.140	0.087	0.088	0.154	0.053	0.051	0.057	**na**					
[25]	Hipposideros cf. ruber	0.084	0.142	0.094	0.089	0.157	0.081	0.081	0.082	0.072	**0.033**				
[26]	*Macronycteris commersoni*	0.155	0.186	0.183	0.167	0.193	0.163	0.162	0.161	0.168	0.174	**0.012**			
[27]	*Macronycteris cryptovalorona*	0.149	0.176	0.170	0.163	0.185	0.161	0.161	0.158	0.160	0.162	0.028	**0.003**		
[28]	*Macronycteris gigas*	0.153	0.179	0.178	0.169	0.190	0.165	0.165	0.161	0.165	0.169	0.026	0.029	**0.012**	
[29]	*Macronycteris vittata*	0.150	0.181	0.180	0.168	0.192	0.158	0.159	0.158	0.159	0.164	0.026	0.029	0.027	**0.006**

Maximum likelihood and Bayesian phylogenies from a 452-individual alignment of cyt-b are shown in Suppl. material 2: Figures S2, Suppl. material 3: Figures S3. Identical haplotypes were pruned from this tree to produce the 303 unique-haplotype alignment shown in Figure 2. The 303 haplotype alignment used in the ML and BI gene tree analyses ranged from 413 to 1140 base pairs (bp) in length (89.9% complete matrix). Only the Bayesian topology is shown, but both posterior probabilities and bootstrap values are depicted at common, well supported nodes. Multiple, geographically cohesive clades are evident for the three widely distributed Afrotropical *Hippposideros*, *H. beatus*, *H. caffer*, and *H. ruber*.

**Figure 2.**
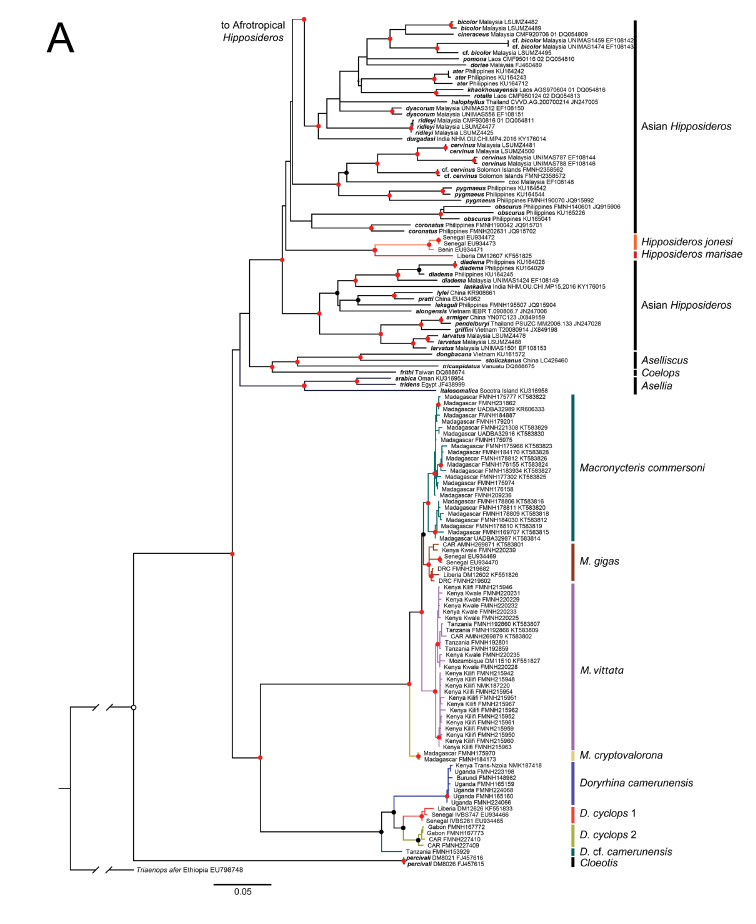
Parts **A** and **B**. Phylogeny of Hipposideridae based on Bayesian analysis of 303 cyt-b sequences. Colored lines denote well supported clades and symbols denote nodal support: red circles, BS ≥ 70%, PP ≥ 0.95; black circles BS ≥ 70%, PP ≤ 0.95; open circles BS ≤ 70%, PP ≥ 0.95.

**Figure 2.**
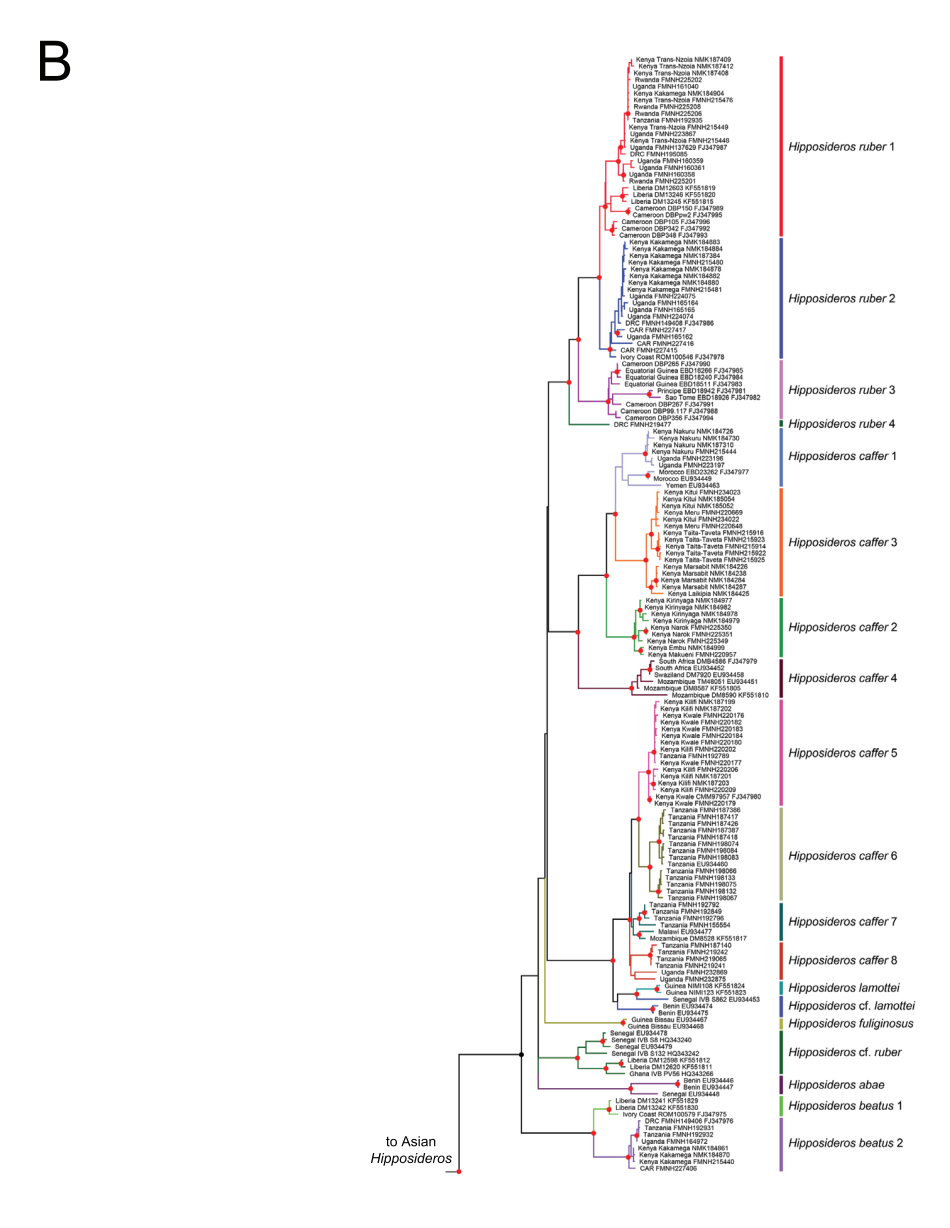
Continued.

**Figure 3.**
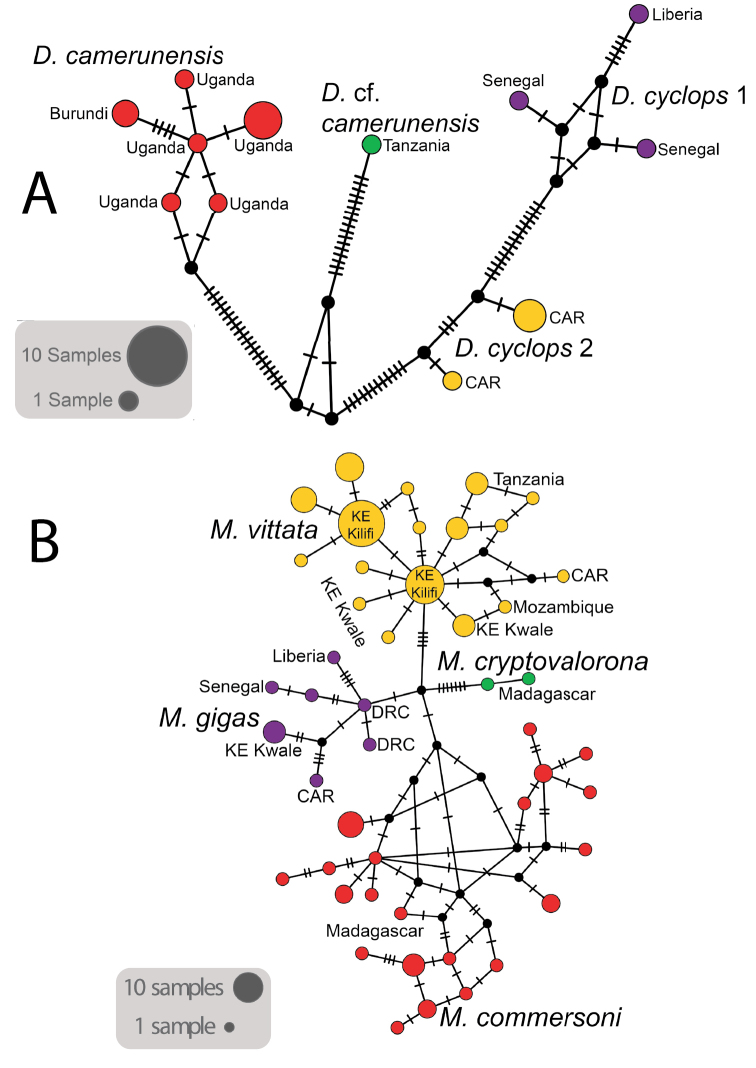
Substitution network plots for Afrotropical hipposiderids **A***Doryrhina***B***Macronycteris*.

**Figure 4.**
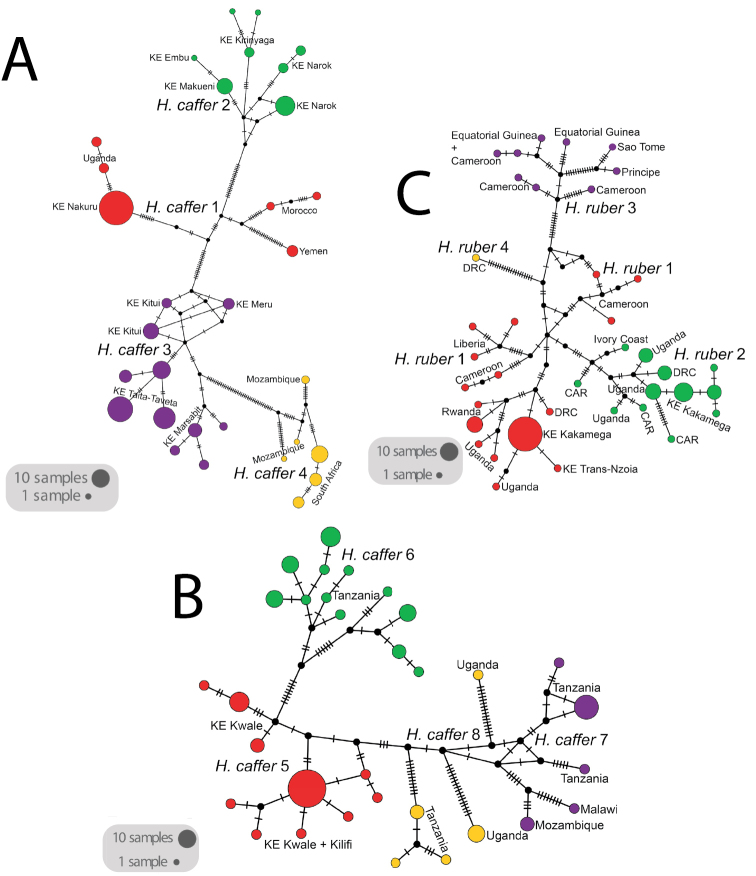
Substitution network plots for Afrotropical hipposiderids **A***Hipposideros caffer* clades 1–4 **B***Hipposideros caffer* clades 5–8 **C***H. ruber* clades.

Substitution networks for cyt-b haplotypes for *Doryrhina*, *Macronycteris*, and *Hipposideros* are shown in Figures 3, 4, showing the genetic and geographic relationships of the clades identified in Figure 2.

**Figure 5.**
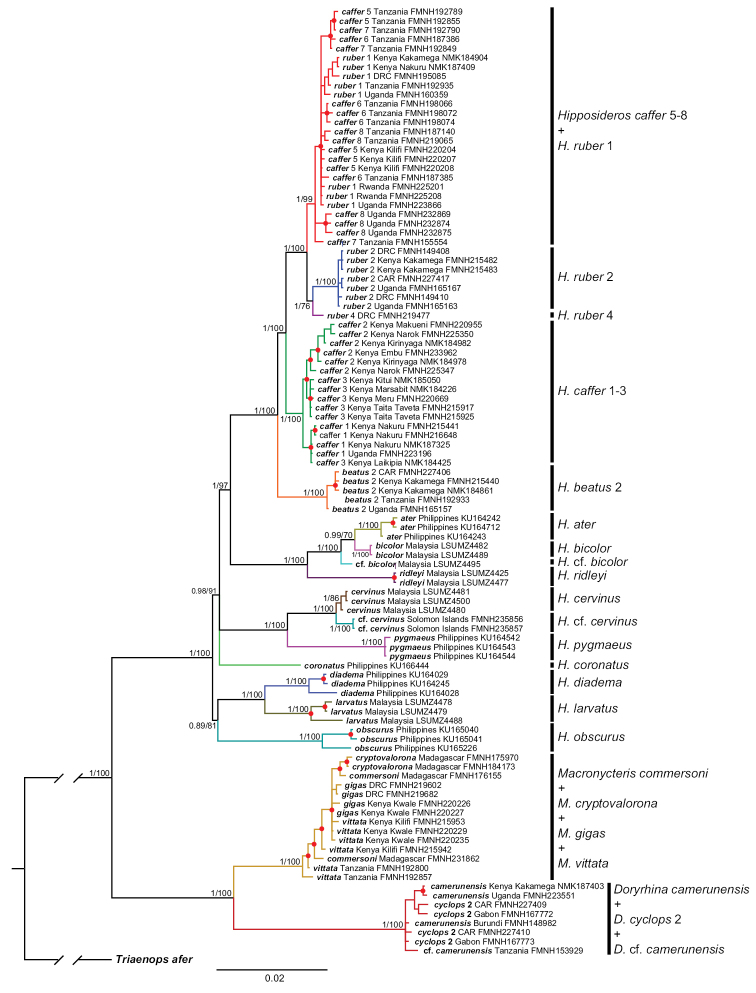
Phylogeny of Hipposideridae based on Bayesian analysis of 103 concatenated nuclear intron sequences. Numbers denote posterior probabilities (BI) and bootstrap percentages (ML); red circles at more terminal nodes indicate BS ≥ 70%, PP ≥ 0.95.

Maximum likelihood and Bayesian phylogenies from a 103-individual alignment of four concatenated introns for *Doryrhina*, *Macronycteris*, and *Hipposideros* are shown in Figure 5. Many of the numbered clades in Figures 2–4 are jumbled in Figure 5; they are not recovered as monophyletic units and the geographic structure evident in mtDNA analyses disappears.

**Figure 6.**
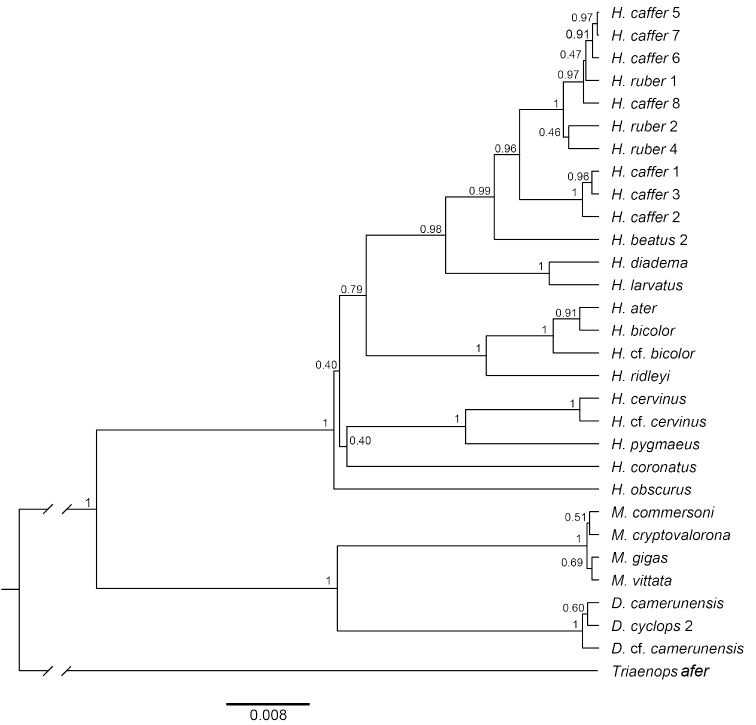
Species tree Hipposideridae based on StarBEAST analysis of four introns. Posterior probabilities appear at all nodes.

A species tree generated using StarBEAST from the four introns appears in Figure 6. It depicts well-supported relationships among the various clades allied with *H. caffer*, *H. ruber*, and *H. beatus*. Remarkably, and in contrast with the concatenated analyses, it shows support for the Asian dyad *H. diadema* and *H. larvatus* as sister to these *ruber* subgroup members, with the Asian *ater* subgroup outside this pairing. There is little support for the deeper phylogenetic nodes.

## Discussion

### Overall genetic variability

The three Afrotropical hipposiderid genera differ substantially in terms of their internal genetic differentiation. Clades of *Hipposideros* are separated by cyt-b *p*-distances averaging 9.7% (2.5–16.1%), whereas *Doryrhina* clades average p-distances of 4.8% (3.0–5.7%) and *Macronycteris* clades 2.7% (2.6–2.9%). Distance values for these genera tend to fall at the lower end of values obtained with similar sampling intensity for species-ranked clades in other Afrotropical bat genera: 2.5% for *Otomops* (Patterson et al. 2018), 9.3% for *Miniopterus* (Demos et al. 2020), 10% for *Scotophilus* and *Rhinolophus* (Demos et al. 2018, 2019a), 13.5% for *Myotis* (Patterson et al. 2019), and 17% for *Nycteris* (Demos et al. 2019b). Fewer cyt-b substitutions on average for these hipposiderids does not limit support for individual clades, and because distances do not approach those characteristic of substitutional saturation, the cyt-b tree recovers much of the deeper phylogenetic structure evident with nuclear intron sequences (compare Figs 2, 5).

### Phylogenetics

Both cyt-b and intron analyses securely recovered *Doryrhina*, *Macronycteris*, and *Hipposideros* as monophyletic. *Doryrhina + Macronycteris* are sister to the remaining hipposiderids. However, only the cyt-b analysis included the hipposiderid genera *Aselliscus*, *Coelops*, and *Asellia* alongside *Hipposideros*. That analysis recovered all four genera as monophyletic with strong support. *Aselliscus* and *Coelops* were recovered as sister to *Hipposideros*, with *Asellia* joining later, but these relationships lacked confident support.

Using a supermatrix approach on exemplars of 46 species of hipposiderids, Amador et al. (2018) found *Hipposideros* sensu stricto to be paraphyletic. They recovered a mostly Asian group of *Hipposideros* as sister to two subclades, *Coelops + Aselliscus* and *Asellia* + African hipposiderids excluding *H. jonesi*, which was recovered with the Asian taxa. Paraphyly in this molecular analysis echoed earlier indications of *Hipposideros* paraphyly from morphology (Bogdanowicz and Owen 1998; Hand and Kirsch 1998, 2003). In another supermatrix analysis of exemplars belonging to 49 hipposiderid species, Shi and Rabosky (2015) failed to recover *Macronycteris* as monophyletic; *M. commersoni* was sister to all remaining hipposiderids, but strangely it did not group with *M. gigas*. When the anomalous position of *M. commersoni* in their tree is ignored, their topology is highly similar to that of Figure 2, except that *Asellia* (*Aselliscus*, *Coelops*) become the sister of *Hipposideros* (*Macronycteris*, *Doryrhina*), rather than sister of just *Hipposideros*. Using both mitochondrial and nuclear loci, Lavery et al. (2014) found that 17 species of Asian, Oceanian and Australasian *Hipposideros* were monophyletic with respect to the genera *Aselliscus*, *Coelops*, and *Anthops*. Clearly, missing data and missing taxa compromise all of these phylogenetic appraisals, so that the question of hipposiderid and *Hipposideros* monophyly remains open. However, subject to its sampling limitations, there is clear support in our analyses of monophyly for *Doryrhina*, *Macronycteris*, and *Hipposideros* as we apply these names.

Despite employing different mitochondrial and nuclear loci and using different sets of taxa, the phylogeny recovered by Lavery et al. (2014) is largely congruent with that in Figure 5. Their earliest diverging species group of *Hipposideros* is the *calcaratus* group, not represented in our tree unless *H. obscurus* is a member (Table 1). Their next diverging unit is the *diadema* group, which is also positioned near the base of our tree. Their other two groups are paired: the *galeritus* group (which includes *H. cervinus*, indicating that this species is misclassified as *calcaratus* member) joined with the *bicolor*/*ater* group. In our intron analysis (Fig. 5), members of the *larvatus* and *diadema* groups join *H. obscurus* as sister to all remaining *Hipposideros* groups. The remainder form a trichotomy: *H. coronatus*, typically considered in the *bicolor* group; *H. pygmaeus* and *H. cervinus*, which are listed in different groups but were both considered members of the *galeritus* unit by Tate (1941); and the erstwhile *bicolor* group (sensu Hill 1963), which was subdivided into the *ater* subgroup (for Asian, Oceanian, and Australasian species) and the *ruber* subgroup (for Atrotropical ones) by Monadjem (2019).

The *ater* subgroup members included in our mitochondrial analysis (Fig. 2) form a well-supported clade consisting of *H. bicolor*, *H. cineraceus*, *H. pomona*, *H. doriae*, *H. ater*, *H. khaokhouayensis*, *H. rotalis*, *H. halophyllusH. dyacorum*, *H. ridley*, and *H. durgadasi*. This group is sister to all analyzed members of the *ruber* subgroup: the various clades allied with *Hipposideros beatus*, *H. caffer*, and *H. ruber*, as well as individuals of the Afrotropical species *H. lamottei* and *H. fuliginosus*. *H. abae*, which was previously considered in the *speoris* group (Simmons 2005; Murray et al. 2012), is clearly a member of the *ruber* group. Outside this pairing are the Asian species *H. cervinus*, *H. coronatus*, *H. coxi*, *H. obscurus*, and *H. pygmaeus*. Two Afrotropical species also lie outside the *ruber + ater* clade: *H. jonesi* and *H. marisae*, both thought to belong to the *speoris* group, appear as sisters in Figure 2A.

Parsimony, topological position, and the strong support of branching relationships in the mitochondrial and intron trees (Fig. 5; also Lavery et al. 2014) make it clear that the Afrotropical *ruber* group represents a comparatively recent colonization event from Asian ancestors–the *ruber* group is sister to the *ater* group and this pair has Asian sisters. However, although the basal dichotomy within *Hipposideros* includes an all Asian clade, lack of support for its sister(s) clouds the phylogenetic position of the *H. jonesi-H. marisae* clade–possibly sister to all sampled *Hipposideros* but more likely sandwiched between Asian clades. In any case, Figure 2 suggests that the *H. jonesi-H. marisae* clade resulted from an earlier African-Asian colonization event.

The lack of agreement in the phylogenetic position of *H. diadema* and *H. larvatus* between the concatenated intron tree (Fig. 5) and the species tree (Fig. 6) deserves comment, as both analyses were based on the same genetic dataset. The position of *H. diadema*-*H. larvatus* as sister to the *ruber* group (Fig. 6) runs counter to both our other genetic analyses (Figs 2, 5) and morphological assessments (Hill 1963; Murray et al. 2012; Table 1). This discrepancy is likely due to the generally weaker support for deep nodes within the tree; in the absence of saturation, this is often taken as evidence of rapid evolutionary radiations (e.g., Almeida et al. 2011). Lanier and Knowles (2014) used simulated data on deep phylogenies to show that species-tree methods do account for coalescent variance at deep nodes but that mutational variance among lineages poses the primary challenge for accurate reconstruction. In either case, vastly expanded genetic sampling via NGS techniques offers the most plausible avenues to clearer resolution.

However, the highly distinctive species *H. megalotis* belongs to its own species group (Table 1) and has not been included in any genetic analysis. Distributed in the Horn of Africa and the Arabian Peninsula, *H. megalotis* is the only hipposiderid with a fold of skin joining the base of the ear pinnae. Its uniquely specialized auditory system and derived dentition (e.g., loss of anterior premolars and enlargement of outer lower incisors), led Hill (1963) to regard it as a species that diverged early from the other groups of African *Hipposideros*. Including this species in future analyses would shed light on the group’s biogeography. Were there three colonizations of Africa by Asian groups of *Hipposideros* or could *H. megalotis* be sister to all Asian lineages of this genus? This information would greatly clarify ancestral geographic range inference.

### Species limits

The lineage delimitation analyses indicate that a number of hipposiderid lineages are either unnamed or unidentified, and also that a number of recognized species may not be genetically and evolutionarily independent.

Previous studies had indicated that both *Hipposideros caffer* (Vallo et al. 2008) and *H. ruber* (Vallo et al. 2011) appear to be complexes of cryptic species. The two are traditionally distinguished on the basis of size and pelage color, *H. ruber* being the larger and more brightly colored form, but this distinction is clouded by geographic variation in size and the presence of both reddish and gray-brown phases in both species. Our mitochondrial analyses identified four *H. ruber* lineages and eight *H. caffer* lineages in two distinct groupings among the sampled populations (Fig. 2). Four of the *caffer* lineages and three of the *ruber* clades were identified as putative species by the BPP analyses (Table 5). The large number of clades in East Africa is remarkable: Kenya and Tanzania each support four of the eight clades allied with *H. caffer*, and all but one of the eight clades known from throughout the continent occur in one or the other East African country. This undoubtedly reflects the region’s great landscape diversity, where West and Central African rainforests reach their eastern limit, southern savannas reach their northern limits, the Sahel reaches its southern limits, and all are riven by the African Rift Valley. It also is a product of our sampling intensity (see Suppl. material 1: Fig. S1).

**Table 5.** Lineage delimitation results from BPP based on the four intron dataset for mtDNA-supported clades of Afrotropical Hipposideridae. PS1-PS4 refer to four different prior schemes based on population size and age of divergence priors (see Table 3 for parameter details). Bold font indicates that the putative species was delimited under all parameter settings.

Putative Species	PS1	PS2	PS3	PS4
*Doryrhina camerunensis*	0.30	0.76	0.95	0.51
D. cf. camerunensis	0.32	0.73	0.97	0.79
*D. cyclops* 2	0.23	0.68	0.95	0.51
***Hipposideros beatus* 2**	**1**	**1**	**1**	**1**
***H. caffer* 1**	**0.99**	**0.99**	**0.99**	**0.99**
***H. caffer* 2**	**0.99**	**1**	**1**	**1**
***H. caffer* 3**	**0.99**	**0.99**	**0.99**	**0.99**
*H. caffer* 5	0.14	0.18	0.11	0.08
*H. caffer* 6	0.56	0.61	0.85	0.82
*H. caffer* 7	0.14	0.18	0.11	0.08
***H. caffer* 8**	**0.99**	**0.99**	**0.99**	**0.99**
***H. ruber* 1**	**0.99**	**0.99**	**0.99**	**0.99**
***H. ruber* 2**	**1**	**0.99**	**1**	**0.99**
***H. ruber* 4**	**0.99**	**0.99**	**0.99**	**0.99**
*Macronycteris commersoni*	0.24	0.72	0.94	0.52
*M. cryptovalorona*	0.35	0.81	0.97	0.76
*M. gigas*	0.09	0.43	0.91	0.38
*M. vittata*	0.11	0.44	0.90	0.34

Because some cyt-b sequences were used in multiple studies of this group, it is possible to relate our clade labels to those used by earlier studies (Table 6). Based on attributions made on morphological grounds by Vallo et al. (2008) and Monadjem et al. (2013), some well-supported but unnamed clades in our analysis can be identified. For instance, *caffer*1 has a distributional range and includes specimens previously identified as *Hipposideros tephrus* (Appendix I), while specimens of *caffer*4 come from near the type locality of *H. caffer* Sundevall, 1846, and may well represent that species. However, no samples confidently identified as *H. ruber* from the vicinity of its type locality have been sequenced, leaving the application of that name to clades in any of these trees purely conjectural. Applying formal names only after integrative taxonomic assessment is a responsible course as multispecies coalescent models like BPP can lead to over-splitting of species, especially when applied to geographically variable species complexes with parapatric distributions (Chambers and Hillis 2020).

**Table 6.** Clade names and associated binomials (if used) for three analyses of cryptic lineages within the *ruber* species group of *Hipposideros*. No genetic analysis of this group has included type material; consequently, the application of binomials hinges on the robustness of ancillary morphological analyses, which were not conducted in our study. Boldfaced names denote clades supported by all four prior schemes in our BPP delimitation analyses.

Vallo et al. (2008)		Monadjem et al. (2013)		This paper
A1	*H. caffer*			caffer 4
		A1a	*H. caffer*	caffer 4
		A1b	*H. caffer*	caffer 4
A2	*H. caffer*		*H. caffer tephrus*	**caffer 1**
B	*H. ruber*			caffer 5
		B1	*H. ruber*	cf. lamottei
		B2	*H. ruber*	caffer 7
C1	*H. ruber*			**ruber 1**, **ruber 2**
		C1a	*H. cf. ruber*	**ruber 1**
		C1b	*H. cf. ruber*	**ruber 1**
C2	*H. ruber*	C2	*H. cf. ruber*	ruber 3
				ruber 4
D	*H. ruber*		*H. cf. ruber*	cf. ruber
		D1	*H. cf. ruber*	cf. ruber
		D2	*H. cf. ruber*	cf. ruber
–		E1	*H. cf. ruber*	cf. ruber
–		E2	*H. cf. ruber*	cf. ruber
–		–		**caffer 2**
–		–		**caffer 3**
–		–		caffer 6
–		–		**caffer 8**
abae	*H. abae*	abae	*H. abae*	abae
beatus	*H. beatus*	beatus	*H. beatus*	beatus1, **beatus 2**
fuliginosus	*H. fuliginosus*	fuliginosus	*H. fuliginosus*	fuliginosus
lamottei	*H. lamottei*	lamottei	*H. lamottei*	lamottei

*Doryrhina* is a poorly known genus characterized morphologically by the peculiar club-shaped processes on the central and posterior nose leaves. This trait is shared by the two recognized African species, *D. cyclops* and *D. camerunensis*, which differ chiefly in size (the latter is larger, with forearm lengths >75 mm). Although *D. cyclops* is considered to be monotypic, mitochondrial sequences clearly separate West African populations in Liberia and Senegal (*cyclops*1) from Central African populations in Gabon and Central African Republic (*cyclops*2), and these are substantially separated from *D. camerunensis* and a specimen referred to that species from Tanzania (Figs 2, 3). However, both the intron analysis (Fig. 5) and the species tree (Fig. 6) show little or no geographic structure. The BPP analyses confirm that none of the mitochondrial clades is behaving as an independent evolutionary lineage (Table 5). Geographic structure in mtDNA but continent-wide admixture in the nuclear genome could result from either male-biased dispersal with female philopatry or highly structured seasonal migrations, which are known in other hipposiderids. In any case, the genetic patterns of *Doryrhina* are hard to reconcile with its space-use behavior; individuals appear to have very small home ranges, on the order of a few hectares (Monadjem 2019). An integrative taxonomic review of the genus *Doryrhina* is needed to determine the validity of *D. cyclops* and *D. camerunensis*. It would also shed light on whether six Australo-Papuan species tentatively allocated to that genus (cf. Monadjem 2019) belong there or elsewhere. Tate (1941) had earlier allocated those species to the Australasian *muscinus* group, convergent on but separate from his Afrotropical *cyclops* group, but Hill (1963) later united these groups.

Our analysis included four of the five recognized species of *Macronycteris*, lacking only *M. thomensis*, which is endemic to São Tomé Island in the Gulf of Guinea. Two species, *M. gigas* and *M. vittata*, occur on the African mainland and two others, *M. commersoni* and *M. cryptovalorona*, occur on Madagascar. *Macronycteris cryptovalorona* was named only in 2016, on the basis of its strong genetic divergence from *M. commersoni*; it appears in Figure 2 as sister to all three remaining species of *Macronycteris*. Despite a search for diagnostic characters, Goodman et al. (2016) could not distinguish it morphologically from *M. commersoni*. Both species are known to occur in the same caves in south central and southwestern Madagascar (Goodman et al. 2016; Rakotoarivelo et al. 2019). On the other hand, *M. vittata* and *M. gigas* are distinguished typically on the basis of size and pelage color (cf. Monadjem 2019). They are also known to occur together in the same cave (Shimoni Cave in Kwale, Kenya; Webala et al. 2019), where they utilize echolocation calls with different peak frequencies: *vittata* at 64–70 kHz and *gigas* at 53.4–54.8 kHz. Both in Africa and on Madagascar, these pairs of taxa appear to act as distinct species, but the monophyly evident in the cyt-b sequences (Figs 2, 4) disappears in the nuclear intron analyses. BPP analyses fail to resolve any of the *Macronycteris* species, and none appear as monophyletic in the concatenated intron analyses.

Our results clearly underscore the importance of using multilocus datasets to evaluate phylogenetic and phylogeographic relationships at the genus and species level in mammals. Use of a single genetic system may lead to widely divergent conclusions regarding species identity and distribution. Toews and Brelsford (2012) reviewed cases of mito-nuclear discordance in animals generally. Fully 18% of the cases they reviewed had discordant patterns of mitochondrial and nuclear DNA. In most cases, such patterns are attributable to adaptive introgression of mtDNA, demographic disparities, and sex-biased asymmetries; in some cases they found evidence for hybrid zone movement or human agency. Discordant patterns of variation between mitochondrial and nuclear DNA have been reported in at least six other families of bats (Nesi et al. 2011; Furman et al. 2014; Naidoo et al. 2016; Hassanin et al. 2018; Demos et al. 2019a; Gürün et al. 2019). Gürün et al. (2019) implicated the role of sex-biased dispersal in causing such discordance, male dispersal spreading nuclear variation farther and faster than the movement of mitochondria. This may be a more general pattern in bats (see also Demos et al. 2019b). To understand the processes responsible for these discordant patterns of genome evolution, extensive genomic sampling and far fuller knowledge of natural history will be required.

## Appendix I

**Table:** Genetic sampling of Hipposideridae. Wherever possible, the voucher numbers associated with the genetic samples are specified. Accession numbers identify sequences downloaded from GenBank or accessioned to Genbank for this study. The designation 'redundant' indicates a cyt-b sequence that was omitted from the 303 individual alignment because of its identity to another. Institutional acronyms are as follows: AMNH – American Museum of Natural History, New York; CM – Carnegie Museum, Pittsburgh; CVVD – ?; DM – Durban Natural Science Museum, Durban; EBD – Estación Biológica de Doñana, Sevilla; FMNH – Field Museum of Natural History, Chicago; IEBR-T – Thong collection at Institute of Ecology and Biological Resources, Hanoi; IVB – Institute of Vertebrate Biology, Brno; KU – Biodiversity Institute and Natural History Museum, University of Kansas, Lawrence; Czech Academy of Sciences, Prague; LSUMZ – Lousiana State University Museum of Natural Science-Mammal Tissues, Baton Rouge; NHMOU – Natural History Museum of Osmania University, Hyderabad; NMK – National Museums of Kenya, Nairobi; NMP – National Museum, Prague; PSUZC – Princess Maha Chakri Sirindhorn Natural History Museum, Songkhla; ROM – Royal Ontario Museum, Toronto; SMF – Senckenberg Museum, Frankfurt; TM – Transvaal Museum, Pretoria; TTU – Texas Tech University Museum, Lubbock; UADBA – Université d'Antananarivo, Département de Biologie Animale, Antananarivo; UNIMAS – University of Malaysia Sarawak Natural History Museum, Kuching.

Voucher	cyt-b	ACOX2	COPS	ROGDI	STAT5	ScientificName	Country	Latitude	Longitude
	KU316954					*Asellia arabica*	Oman	17.100	54.080
	KU316958					*Asellia italosomalica*	Yemen	12.670	54.120
	JF438999					*Asellia tridens*	Libya	24.933	10.167
IEBR-T	KU161572					*Aselliscus dongbacana*	Vietnam	22.360	105.395
	LC426460					*Aselliscus stoliczkanus*	China	
	DQ888675					*Aselliscus tricuspidatus*	Vanuatu	-15.307	166.926
DM 8021	FJ457616					*Cloeotis percivali*	Swaziland	-25.817	31.283
DM 8026	FJ457615					*Cloeotis percivali*	Swaziland	-25.817	31.283
	DQ888674					*Coelops frithi*	Taiwan	21.948	120.780
FMNH 148981	redundant					*Doryrhina camerunensis*	Burundi	-2.100	29.383
FMNH 148982	MT149719	MT149615	MT149513	MT149418	MT149317	*Doryrhina camerunensis*	Burundi	-2.850	29.400
NMK 187403	redundant	MT149616	MT149514	MT149419	MT149318	*Doryrhina camerunensis*	Kenya	0.344	34.857
NMK 187418	MT149720					*Doryrhina camerunensis*	Kenya	0.344	34.857
FMNH 165159	MT149721					*Doryrhina camerunensis*	Uganda	1.683	31.533
FMNH 165160	MT149722					*Doryrhina camerunensis*	Uganda	1.683	31.533
FMNH 223198	MT149723					*Doryrhina camerunensis*	Uganda	0.445	32.889
FMNH 223551	redundant	MT149617	MT149515	MT149420	MT149319	*Doryrhina camerunensis*	Uganda	0.445	32.889
FMNH 224066	MT149724					*Doryrhina camerunensis*	Uganda	0.501	30.426
FMNH 224068	MT149725					*Doryrhina camerunensis*	Uganda	0.501	30.426
FMNH 153929	MT149726	MT149618	MT149516	MT149421	MT149320	Doryrhina cf. camerunensis	Tanzania	-4.942	38.733
DM 12626	KF551833					*Doryrhina cyclops1*	Liberia	7.553	-8.492
IVB S261	EU934465					*Doryrhina cyclops1*	Senegal	12.883	-12.717
IVB S747	EU934466					*Doryrhina cyclops1*	Senegal	13.333	-13.217
FMNH 227409	MT149727	MT149619	MT149517	MT149422	MT149321	*Doryrhina cyclops2*	Central African Republic	13.033	16.410
FMNH 227410	MT149728	MT149620	MT149518	MT149423	MT149322	*Doryrhina cyclops2*	Central African Republic	3.033	16.410
FMNH 167772	MT149729	MT149621	MT149519	MT149424	MT149323	*Doryrhina cyclops2*	Gabon	-2.283	10.497
FMNH 167773	MT149730	MT149622	MT149520	MT149425	MT149324	*Doryrhina cyclops2*	Gabon	-2.283	10.497
NMP 91850	EU934446					*Hipposideros abae*	Benin	7.783	2.267
NMP 91851	EU934447					*Hipposideros abae*	Benin	7.783	2.267
IVB S822	EU934448					*Hipposideros abae*	Senegal	12.350	-12.317
IEBR-T 90806.7	JN247006					*Hipposideros alongensis*	Vietnam	
YN07C123	JX849159					*Hipposideros armiger*	China	23.600	102.002
UNIMAS 729	EF108140 redundant					*Hipposideros ater*	Malaysia	1.407	110.169
UNIMAS 1577	EF108139 redundant					*Hipposideros ater*	Malaysia	3.801	113.785
KU 164242	MT149731	MT149623	MT149521	MT149426	MT149325	*Hipposideros ater*	Philippines	13.796	120.159
KU 164243	MT149732	MT149624	MT149522	MT149427	MT149326	*Hipposideros ater*	Philippines	13.796	120.159
KU 164712	MT149733	MT149625	MT149523	MT149428	MT149327	*Hipposideros ater*	Philippines	19.085	121.241
ROM 100579	FJ347975					*Hipposideros beatus1*	Ivory Coast	6.930	-7.217
DM 13241	KF551829					*Hipposideros beatus1*	Liberia	7.553	-8.492
DM 13242	KF551830					*Hipposideros beatus1*	Liberia	7.553	-8.492
FMNH 227406	MT149734	MT149613	MT149524	MT149429	MT149328	*Hipposideros beatus2*	Central African Republic	3.033	16.410
FMNH 149406	FJ347976					*Hipposideros beatus2*	Democratic Republic of Congo	-1.417	28.583
FMNH 215440	MT149735	MT149626	MT149525	MT149430	MT149329	*Hipposideros beatus2*	Kenya	0.352	34.865
NMK 184861	MT149736	MT149627	MT149526	MT149431	MT149330	*Hipposideros beatus2*	Kenya	0.356	34.861
NMK 184864	redundant					*Hipposideros beatus2*	Kenya	0.360	34.861
NMK 184870	MT149737					*Hipposideros beatus2*	Kenya	0.352	34.865
FMNH 192931	MT149738					*Hipposideros beatus2*	Tanzania	-1.094	31.515
FMNH 192932	MT149739					*Hipposideros beatus2*	Tanzania	-1.094	31.515
FMNH 192933	redundant	MT149628	MT149527	MT149432	MT149331	*Hipposideros beatus2*	Tanzania	-1.094	31.515
FMNH 164972	MT149740					*Hipposideros beatus2*	Uganda	1.733	31.467
FMNH 165157	redundant	MT149629	MT149528	MT149433	MT149332	*Hipposideros beatus2*	Uganda	1.683	31.533
LSUMZ MT-4482	MT149741	MT149630	MT149529	MT149434	MT149333	*Hipposideros bicolor*	Malaysia	1.970	103.500
LSUMZ MT-4489	MT149742	MT149631	MT149530		MT149334	*Hipposideros bicolor*	Malaysia	1.970	103.500
FMNH 215441	redundant	MT149632	MT149531	MT149435	MT149335	*Hipposideros caffer1*	Kenya	-0.346	36.119
FMNH 215442	redundant					*Hipposideros caffer1*	Kenya	-0.346	36.119
FMNH 215443	redundant					*Hipposideros caffer1*	Kenya	-0.346	36.119
FMNH 215444	MT149743					*Hipposideros caffer1*	Kenya	-0.346	36.119
FMNH 215445	redundant					*Hipposideros caffer1*	Kenya	-0.346	36.119
FMNH 215446	redundant					*Hipposideros caffer1*	Kenya	-0.346	36.119
FMNH 215447	redundant					*Hipposideros caffer1*	Kenya	-0.346	36.119
FMNH 216628	redundant					*Hipposideros caffer1*	Kenya	-0.346	36.119
FMNH 216629	redundant					*Hipposideros caffer1*	Kenya	-0.346	36.119
FMNH 216630	redundant					*Hipposideros caffer1*	Kenya	-0.346	36.119
FMNH 216631	redundant					*Hipposideros caffer1*	Kenya	-0.346	36.119
FMNH 216632	redundant					*Hipposideros caffer1*	Kenya	-0.346	36.119
FMNH 216645	redundant					*Hipposideros caffer1*	Kenya	-0.346	36.119
FMNH 216646	redundant					*Hipposideros caffer1*	Kenya	-0.346	36.119
FMNH 216647	redundant					*Hipposideros caffer1*	Kenya	-0.346	36.119
FMNH 216648	redundant	MT149633	MT149532	MT149436	MT149336	*Hipposideros caffer1*	Kenya	-0.346	36.119
FMNH 225346	redundant					*Hipposideros caffer1*	Kenya	-0.346	36.119
FMNH 225747	redundant					*Hipposideros caffer1*	Kenya	-0.564	36.254
FMNH 225748	redundant					*Hipposideros caffer1*	Kenya	-0.564	36.254
FMNH 225749	redundant					*Hipposideros caffer1*	Kenya	-0.564	36.254
FMNH 225750	redundant					*Hipposideros caffer1*	Kenya	-0.564	36.254
FMNH 225751	redundant					*Hipposideros caffer1*	Kenya	-0.564	36.254
NMK 184726	MT149744					*Hipposideros caffer1*	Kenya	-0.564	36.254
NMK 184727	redundant					*Hipposideros caffer1*	Kenya	-0.564	36.254
NMK 184728	redundant					*Hipposideros caffer1*	Kenya	-0.564	36.254
NMK 184729	redundant					*Hipposideros caffer1*	Kenya	-0.564	36.254
NMK 184730	MT149745					*Hipposideros caffer1*	Kenya	-0.564	36.254
NMK 184760	redundant					*Hipposideros caffer1*	Kenya	-0.430	36.174
NMK 184842	redundant					*Hipposideros caffer1*	Kenya	-0.539	36.294
NMK 184843	redundant					*Hipposideros caffer1*	Kenya	-0.539	36.294
NMK 187310	MT149718					*Hipposideros caffer1*	Kenya	-0.539	36.294
NMK 187311	redundant					*Hipposideros caffer1*	Kenya	-0.539	36.294
NMK 187312	redundant					*Hipposideros caffer1*	Kenya	-0.539	36.294
NMK 187323	redundant					*Hipposideros caffer1*	Kenya	-0.346	36.119
NMK 187324	redundant					*Hipposideros caffer1*	Kenya	-0.346	36.119
NMK 187325	redundant	MT149634	MT149533	MT149437	MT149337	*Hipposideros caffer1*	Kenya	-0.346	36.119
NMK 187326	redundant					*Hipposideros caffer1*	Kenya	-0.346	36.119
EBD 23262	FJ347977					*Hipposideros caffer1*	Morocco	30.630	9.830
NMP	EU934449					*Hipposideros caffer1*	Morocco	3.801	113.785
FMNH 223196	MT149746	MT149635	MT149534	MT149438	MT149338	*Hipposideros caffer1*	Uganda	0.445	32.889
FMNH 223197	MT149747					*Hipposideros caffer1*	Uganda	0.445	32.889
NMP	EU934463					*Hipposideros caffer1*	Yemen	15.283	44.167
FMNH 220955	redundant	MT149639	MT149538	MT149441	MT149342	*Hipposideros caffer2*	Kenya	-2.203	37.714
FMNH 220956	redundant					*Hipposideros caffer2*	Kenya	-2.203	37.714
FMNH 220957	MT149753					*Hipposideros caffer2*	Kenya	-2.203	37.714
FMNH 220958	redundant					*Hipposideros caffer2*	Kenya	-2.203	37.714
FMNH 225347	redundant	MT149640	MT149539	MT149442	MT149343	*Hipposideros caffer2*	Kenya	-1.547	35.306
FMNH 225348	redundant					*Hipposideros caffer2*	Kenya	-1.531	35.320
FMNH 225349	MT149754					*Hipposideros caffer2*	Kenya	-1.531	35.320
FMNH 225350	MT149755	MT149641	MT149540	MT149443		*Hipposideros caffer2*	Kenya	-1.531	35.320
FMNH 225351	MT149756					*Hipposideros caffer2*	Kenya	-1.531	35.320
FMNH 225352	redundant					*Hipposideros caffer2*	Kenya	-1.531	35.320
NMK 184977	MT149749					*Hipposideros caffer2*	Kenya	-0.117	34.541
NMK 184978	MT149750	MT149637	MT149536	MT149440	MT149340	*Hipposideros caffer2*	Kenya	-0.117	34.541
NMK 184979	MT149751					*Hipposideros caffer2*	Kenya	-0.117	34.541
NMK 184981	redundant					*Hipposideros caffer2*	Kenya	-0.117	34.541
NMK 184982	MT149752	MT149638	MT149537		MT149341	*Hipposideros caffer2*	Kenya	-0.117	34.541
NMK 184999	MT149748	MT149636	MT149535	MT149439	MT149339	*Hipposideros caffer2*	Kenya	-0.555	37.388
FMNH 215914	MT149768					*Hipposideros caffer3*	Kenya	-3.706	38.776
FMNH 215915	redundant					*Hipposideros caffer3*	Kenya	-3.706	38.776
FMNH 215916	MT149769					*Hipposideros caffer3*	Kenya	-3.706	38.776
FMNH 215917	redundant	MT149646	MT149545	MT149448	MT149348	*Hipposideros caffer3*	Kenya	-3.706	38.776
FMNH 215918	redundant					*Hipposideros caffer3*	Kenya	-3.706	38.776
FMNH 215921	redundant					*Hipposideros caffer3*	Kenya	-3.076	39.217
FMNH 215922	MT149770					*Hipposideros caffer3*	Kenya	-3.076	39.217
FMNH 215923	MT149771					*Hipposideros caffer3*	Kenya	-3.076	39.217
FMNH 215924	redundant					*Hipposideros caffer3*	Kenya	-3.076	39.217
FMNH 215925	MT149772	MT149647	MT149546	MT149449	MT149349	*Hipposideros caffer3*	Kenya	-3.076	39.217
FMNH 220648	MT149766					*Hipposideros caffer3*	Kenya	0.170	38.194
FMNH 220669	MT149767	MT149645	MT149544	MT149447	MT149347	*Hipposideros caffer3*	Kenya	0.024	38.066
FMNH 234022	MT149757					*Hipposideros caffer3*	Kenya	-1.019	38.326
FMNH 234023	MT149758					*Hipposideros caffer3*	Kenya	-0.992	38.330
NMK 184226	MT149762	MT149644	MT149543	MT149446	MT149346	*Hipposideros caffer3*	Kenya	2.320	37.994
NMK 184238	MT149763					*Hipposideros caffer3*	Kenya	2.320	37.994
NMK 184284	MT149764					*Hipposideros caffer3*	Kenya	2.320	37.994
NMK 184287	MT149765					*Hipposideros caffer3*	Kenya	2.283	37.954
NMK 184425	MT149761	MT149643	MT149542	MT149445	MT149345	*Hipposideros caffer3*	Kenya	0.228	37.113
NMK 185050	redundant	MT149642	MT149541	MT149444	MT149344	*Hipposideros caffer3*	Kenya	-0.992	38.330
NMK 185051	redundant					*Hipposideros caffer3*	Kenya	-0.992	38.330
NMK 185052	MT149759					*Hipposideros caffer3*	Kenya	-0.992	38.330
NMK 185053	redundant					*Hipposideros caffer3*	Kenya	-0.992	38.330
NMK 185054	MT149760					*Hipposideros caffer3*	Kenya	-0.992	38.330
DM 8587	KF551805					*Hipposideros caffer4*	Mozambique	-23.205	32.499
DM 8590	KF551810					*Hipposideros caffer4*	Mozambique	-12.182	37.550
TM 48051	EU934451					*Hipposideros caffer4*	Mozambique	-21.517	35.100
DM 11007	KF551806 redundant					*Hipposideros caffer4*	South Africa	-27.596	32.220
	FJ347979					*Hipposideros caffer4*	South Africa	-27.660	32.251
	EU934452					*Hipposideros caffer4*	South Africa	-23.999	31.645
DM 7920	EU934458					*Hipposideros caffer4*	Swaziland	-26.870	31.463
FMNH 215941	redundant					*Hipposideros caffer5*	Kenya	-3.300	39.995
FMNH 220176	MT149780					*Hipposideros caffer5*	Kenya	-4.590	39.331
FMNH 220177	MT149781					*Hipposideros caffer5*	Kenya	-4.590	39.331
FMNH 220178	redundant					*Hipposideros caffer5*	Kenya	-4.590	39.331
FMNH 220179	MT149782					*Hipposideros caffer5*	Kenya	-4.590	39.331
FMNH 220180	MT149783					*Hipposideros caffer5*	Kenya	-4.590	39.331
FMNH 220182	MT149784					*Hipposideros caffer5*	Kenya	-4.082	39.483
FMNH 220183	MT149785					*Hipposideros caffer5*	Kenya	-4.082	39.483
FMNH 220184	MT149786					*Hipposideros caffer5*	Kenya	-4.082	39.483
FMNH 220185	redundant					*Hipposideros caffer5*	Kenya	-4.082	39.483
FMNH 220186	redundant					*Hipposideros caffer5*	Kenya	-4.082	39.483
FMNH 220202	MT149773					*Hipposideros caffer5*	Kenya	-3.300	39.995
FMNH 220203	redundant					*Hipposideros caffer5*	Kenya	-3.300	39.995
FMNH 220204	redundant	MT149648	MT149547	MT149450	MT149350	*Hipposideros caffer5*	Kenya	-3.300	39.995
FMNH 220205	redundant					*Hipposideros caffer5*	Kenya	-3.323	40.042
FMNH 220206	MT149774					*Hipposideros caffer5*	Kenya	-3.323	40.042
FMNH 220207	redundant	MT149649	MT149548	MT149451	MT149351	*Hipposideros caffer5*	Kenya	-3.323	40.042
FMNH 220208	redundant	MT149650	MT149549	MT149452	MT149352	*Hipposideros caffer5*	Kenya	-3.323	40.042
FMNH 220209	MT149775					*Hipposideros caffer5*	Kenya	-3.323	40.042
FMNH 233985	redundant					*Hipposideros caffer5*	Kenya	-3.335	40.031
NMK 187199	MT149776					*Hipposideros caffer5*	Kenya	-3.323	40.042
NMK 187200	redundant					*Hipposideros caffer5*	Kenya	-3.323	40.042
NMK 187201	MT149777					*Hipposideros caffer5*	Kenya	-3.323	40.042
NMK 187202	MT149778					*Hipposideros caffer5*	Kenya	-3.323	40.042
NMK 187203	MT149779					*Hipposideros caffer5*	Kenya	-3.323	40.042
CM 97957	FJ347980					*Hipposideros caffer5*	Kenya	-4.250	39.383
FMNH 192789	MT149787	MT149651	MT149550	MT149453	MT149353	*Hipposideros caffer5*	Tanzania	-4.902	39.688
FMNH 192855	redundant	MT149652	MT149551	MT149454	MT149354	*Hipposideros caffer5*	Tanzania	-4.902	39.688
FMNH 187385	redundant	MT149653	MT149552	MT149455	MT149355	*Hipposideros caffer6*	Tanzania	-8.003	39.762
FMNH 187386	MT149788	MT149654	MT149553	MT149456		*Hipposideros caffer6*	Tanzania	-8.003	39.762
FMNH 187387	MT149789					*Hipposideros caffer6*	Tanzania	-7.993	39.792
FMNH 187388	redundant					*Hipposideros caffer6*	Tanzania	-7.993	39.792
FMNH 187417	MT149790					*Hipposideros caffer6*	Tanzania	-7.891	39.843
FMNH 187418	MT149791					*Hipposideros caffer6*	Tanzania	-7.891	39.843
FMNH 187426	MT149792					*Hipposideros caffer6*	Tanzania	-7.891	39.843
FMNH 187428	redundant					*Hipposideros caffer6*	Tanzania	-7.993	39.792
FMNH 198066	MT149793	MT149655	MT149554	MT149457	MT149356	*Hipposideros caffer6*	Tanzania	-5.878	39.311
FMNH 198067	MT149794					*Hipposideros caffer6*	Tanzania	-5.878	39.311
FMNH 198072	redundant	MT149656	MT149555	MT149458	MT149357	*Hipposideros caffer6*	Tanzania	-6.244	39.320
FMNH 198073	redundant					*Hipposideros caffer6*	Tanzania	-6.244	39.320
FMNH 198074	MT149795	MT149657	MT149556	MT149459	MT149358	*Hipposideros caffer6*	Tanzania	-6.244	39.320
FMNH 198075	MT149796					*Hipposideros caffer6*	Tanzania	-6.244	39.320
FMNH 198076	redundant					*Hipposideros caffer6*	Tanzania	-6.244	39.320
FMNH 198082	redundant					*Hipposideros caffer6*	Tanzania	-6.280	39.451
FMNH 198083	MT149797					*Hipposideros caffer6*	Tanzania	-6.280	39.451
FMNH 198084	MT149798					*Hipposideros caffer6*	Tanzania	-6.280	39.451
FMNH 198131	redundant					*Hipposideros caffer6*	Tanzania	-5.878	39.311
FMNH 198132	MT149799					*Hipposideros caffer6*	Tanzania	-5.878	39.311
FMNH 198133	MT149800					*Hipposideros caffer6*	Tanzania	-5.878	39.311
NMP	EU934460					*Hipposideros caffer6*	Tanzania	-5.998	39.187
NMP	EU934477					*Hipposideros caffer7*	Malawi	-16.033	35.500
DM 8528	KF551817					*Hipposideros caffer7*	Mozambique	-13.401	34.870
DM 8550	KF551816 redundant					*Hipposideros caffer7*	Mozambique	-13.401	34.870
FMNH 155554	MT149801	MT149658	MT149557	MT149460	MT149359	*Hipposideros caffer7*	Tanzania	-8.519	35.904
FMNH 192790	redundant	MT149659	MT149558	MT149461	MT149360	*Hipposideros caffer7*	Tanzania	-4.902	39.688
FMNH 192792	MT149802					*Hipposideros caffer7*	Tanzania	-5.367	39.645
FMNH 192793	redundant					*Hipposideros caffer7*	Tanzania	-5.367	39.645
FMNH 192794	redundant					*Hipposideros caffer7*	Tanzania	-5.367	39.645
FMNH 192795	redundant					*Hipposideros caffer7*	Tanzania	-5.367	39.645
FMNH 192796	MT149803					*Hipposideros caffer7*	Tanzania	-5.367	39.645
FMNH 192849	MT149804	MT149660	MT149559	MT149462	MT149361	*Hipposideros caffer7*	Tanzania	-4.902	39.688
FMNH 187140	MT149805	MT149661	MT149560	MT149463	MT149362	*Hipposideros caffer8*	Tanzania	-3.798	36.069
FMNH 219065	MT149806	MT149662	MT149561	MT149464	MT149363	*Hipposideros caffer8*	Tanzania	-8.037	34.502
FMNH 219241	MT149807					*Hipposideros caffer8*	Tanzania	-8.037	34.502
FMNH 219242	MT149808					*Hipposideros caffer8*	Tanzania	-7.707	34.031
FMNH 232868	redundant					*Hipposideros caffer8*	Uganda	2.240	31.688
FMNH 232869	MT149809	MT149663	MT149562	MT149465	MT149364	*Hipposideros caffer8*	Uganda	2.240	31.688
FMNH 232874	redundant	MT149664	MT149563	MT149416	MT149365	*Hipposideros caffer8*	Uganda	2.240	31.688
FMNH 232875	MT149810	MT149665	MT149564		MT149366	*Hipposideros caffer8*	Uganda	2.240	31.688
LSUMZ MT-4480	redundant	MT149666		MT149466	MT149367	*Hipposideros cervinus*	Malaysia	1.970	103.500
LSUMZ MT-4481	MT149811	MT149667	MT149565	MT149467	MT149368	*Hipposideros cervinus*	Malaysia	1.970	103.500
LSUMZ MT-4500	MT149812	MT149668	MT149566	MT149468	MT149369	*Hipposideros cervinus*	Malaysia	1.970	103.500
UNIMAS 787	EF108144					*Hipposideros cervinus*	Malaysia	3.316	113.125
UNIMAS 788	EF108146					*Hipposideros cervinus*	Malaysia	3.316	113.125
LSUMZ MT-4495	MT149813	MT149669		MT149469	MT149370	Hipposideros cf. bicolor	Malaysia	1.970	103.500
UNIMAS 1459	EF108142					Hipposideros cf. bicolor	Malaysia	1.716	110.467
UNIMAS 1474	EF108143					Hipposideros cf. bicolor	Malaysia	1.716	110.467
FMNH 235856	MT149814	MT149670	MT149567	MT149470	MT149371	Hipposideros cf. cervinus	Solomon Islands	-10.569	161.913
FMNH 235857	MT149815	MT149671	MT149568	MT149471	MT149372	Hipposideros cf. cervinus	Solomon Islands	-10.569	161.913
NMP 91848	EU934474					Hipposideros cf. lamottei	Benin	7.783	2.267
NMP 91849	EU934475					Hipposideros cf. lamottei	Benin	7.783	2.267
IVB S862	EU934453					Hipposideros cf. lamottei	Senegal	12.350	-12.317
IVB PV56	HQ343266					Hipposideros cf. ruber	Ghana	7.668	-1.962
DM 12598	KF551812					Hipposideros cf. ruber	Liberia	7.553	-8.492
DM 12620	KF551811					Hipposideros cf. ruber	Liberia	7.553	-8.492
IVB S119	EU934478					Hipposideros cf. ruber	Senegal	13.050	-13.083
IVB S132	HQ343242					Hipposideros cf. ruber	Senegal	14.071	-12.572
IVB S1374	EU934479					Hipposideros cf. ruber	Senegal	13.250	-13.217
IVB S8	HQ343240					Hipposideros cf. ruber	Senegal	12.884	-12.755
LSUMZ MT-4423	DQ054809					*Hipposideros cineraceus*	Malaysia	3.717	102.167
FMNH 190042	JQ915701					*Hipposideros coronatus*	Philippines	9.097	125.705
FMNH 202631	JQ915702					*Hipposideros coronatus*	Philippines	9.764	124.266
KU 166444		MT149672		MT149472	MT149373	*Hipposideros coronatus*	Philippines	11.813	125.278
	EF108148					*Hipposideros coxi*	Malaysia	1.378	110.120
	EF108147 redundant					*Hipposideros coxi*	Malaysia	1.378	110.120
UNIMAS 1424	EF108149					*Hipposideros diadema*	Malaysia	5.531	118.072
KU 164028	MT149816	MT149673	MT149569	MT149473	MT149374	*Hipposideros diadema*	Philippines	19.331	121.439
KU 164029	MT149817	MT149674	MT149570	MT149474	MT149375	*Hipposideros diadema*	Philippines	19.331	121.439
KU 164245	MT149818	MT149675	MT149571	MT149475		*Hipposideros diadema*	Philippines	13.796	120.159
	FJ460489					*Hipposideros doriae*	Malaysia	1.117	110.217
NHMOU.CHI MP4.2016	KY176014					*Hipposideros durgadasi*	India	23.317	78.414
UNIMAS 312	EF108150					*Hipposideros dyacorum*	Malaysia	4.401	117.889
UNIMAS 556	EF108151					*Hipposideros dyacorum*	Malaysia	3.316	113.125
	EU934468					*Hipposideros fuliginosus*	Guinea Bissau	11.117	-14.933
	EU934467					*Hipposideros fuliginosus*	Guinea Bissau	11.333	-13.900
	JX849198					*Hipposideros griffini*	Vietnam		
CVVD AG	200700214	JN247005					*Hipposideros halophyllus*	Thailand		
NMP 91842	EU934471					*Hipposideros jonesi*	Benin	7.783	2.267
IVB S804	EU934472					*Hipposideros jonesi*	Senegal	12.350	-12.317
	EU934473					*Hipposideros jonesi*	Senegal	12.350	-12.317
EBD 23514	DQ054816					*Hipposideros khaokhouayensis*	Laos	18.433	102.950
	KF551824					*Hipposideros lamottei*	Guinea	7.570	-8.471
	KF551823					*Hipposideros lamottei*	Guinea	7.570	-8.471
NHMOU.CHI MP15.2016	KY176015					*Hipposideros lankadiva*	India	23.317	78.414
LSUMZ MT-4478	MT149819	MT149676	MT149572	MT149476	MT149376	*Hipposideros larvatus*	Malaysia	1.970	103.500
LSUMZ MT-4479	redundant	MT149677	MT149573		MT149377	*Hipposideros larvatus*	Malaysia	1.970	103.500
LSUMZ MT-4488	MT149820	MT149678	MT149574	MT149477	MT149378	*Hipposideros larvatus*	Malaysia	1.970	103.500
UNIMAS 1485	EF108152 redundant					*Hipposideros larvatus*	Malaysia	1.717	110.467
UNIMAS 1501	EF108153					*Hipposideros larvatus*	Malaysia	1.717	110.467
FMNH 195507	JQ915904					*Hipposideros lekaguli*	Philippines	16.314	121.394
	KR908661					*Hipposideros lylei*	China	25.603	99.752
DM 12607	KF551825					*Hipposideros marisae*	Liberia	7.553	-8.492
FMNH 140601	JQ915906					*Hipposideros obscurus*	Philippines	13.767	124.350
KU 165040	redundant	MT149679	MT149575		MT149379	*Hipposideros obscurus*	Philippines	11.434	122.079
KU 165041	MT149821	MT149680	MT149576	MT149478	MT149380	*Hipposideros obscurus*	Philippines	11.434	122.079
KU 165226	MT149717		MT149577	MT149479	MT149381	*Hipposideros obscurus*	Philippines	13.447	120.426
PSUZC MM2006.129	JN247029					*Hipposideros pendelburyi*	Thailand	7.565	99.624
	DQ054810					*Hipposideros pomona*	Laos	18.250	104.517
	EU434952					*Hipposideros pratti*	China	27.729	115.734
FMNH 190070	JQ915992					*Hipposideros pygmaeus*	Philippines	9.097	125.705
KU 164542	MT149822	MT149681	MT149578	MT149480	MT149382	*Hipposideros pygmaeus*	Philippines	14.823	121.968
KU 164543	redundant	MT149682	MT149579	MT149481	MT149383	*Hipposideros pygmaeus*	Philippines	14.823	121.968
KU 164544	MT149716	MT149683	MT149580	MT149482	MT149384	*Hipposideros pygmaeus*	Philippines	14.823	121.968
LSUMZ MT-4425	MT149715	MT149684	MT149581	MT149483	MT149385	*Hipposideros ridleyi*	Malaysia	3.557	102.761
LSUMZ MT-4477	MT149823		MT149582	MT149484	MT149386	*Hipposideros ridleyi*	Malaysia	3.557	102.761
SMF 83828	DQ054811					*Hipposideros ridleyi*	Malaysia	3.717	102.167
	DQ054813					*Hipposideros rotalis*	Laos	18.250	104.517
	FJ347996					*Hipposideros ruber1*	Cameroon	3.150	13.000
	FJ347995					*Hipposideros ruber1*	Cameroon	4.451	11.571
	FJ347993					*Hipposideros ruber1*	Cameroon	3.564	13.408
	FJ347992					*Hipposideros ruber1*	Cameroon	3.564	13.408
	FJ347989					*Hipposideros ruber1*	Cameroon	5.385	11.688
FMNH 195085	MT149824	MT149685	MT149583	MT149485	MT149387	*Hipposideros ruber1*	D. R. Congo	-4.991	29.080
FMNH 215448	MT149826					*Hipposideros ruber1*	Kenya	1.036	34.753
FMNH 215449	MT149827					*Hipposideros ruber1*	Kenya	1.036	34.753
FMNH 215450	redundant					*Hipposideros ruber1*	Kenya	1.036	34.753
FMNH 215451	redundant					*Hipposideros ruber1*	Kenya	1.036	34.753
FMNH 215452	redundant					*Hipposideros ruber1*	Kenya	1.036	34.753
FMNH 215453	redundant					*Hipposideros ruber1*	Kenya	1.036	34.753
FMNH 215476	MT149828					*Hipposideros ruber1*	Kenya	1.036	34.753
FMNH 215477	redundant					*Hipposideros ruber1*	Kenya	1.036	34.753
FMNH 215478	redundant					*Hipposideros ruber1*	Kenya	1.036	34.753
NMK 184904	MT149825	MT149686	MT149584	MT149486		*Hipposideros ruber1*	Kenya	0.212	34.899
NMK 184905	redundant					*Hipposideros ruber1*	Kenya	0.212	34.899
NMK 187407	redundant					*Hipposideros ruber1*	Kenya	1.036	34.753
NMK 187408	MT149829					*Hipposideros ruber1*	Kenya	1.036	34.753
NMK 187409	MT149830	MT149687	MT149585	MT149487	MT149388	*Hipposideros ruber1*	Kenya	1.036	34.753
NMK 187410	redundant					*Hipposideros ruber1*	Kenya	1.036	34.753
NMK 187412	MT149831					*Hipposideros ruber1*	Kenya	1.036	34.753
DM 12603	KF551819					*Hipposideros ruber1*	Liberia	7.553	-8.492
DM 13245	KF551815					*Hipposideros ruber1*	Liberia	7.553	-8.492
DM 13246	KF551820					*Hipposideros ruber1*	Liberia	7.553	-8.492
FMNH 225201	MT149832	MT149688	MT149586	MT149488	MT149389	*Hipposideros ruber1*	Rwanda	-2.485	29.199
FMNH 225202	MT149833					*Hipposideros ruber1*	Rwanda	-1.504	29.613
FMNH 225203	redundant					*Hipposideros ruber1*	Rwanda	-1.504	29.613
FMNH 225204	redundant					*Hipposideros ruber1*	Rwanda	-1.506	29.615
FMNH 225205	redundant					*Hipposideros ruber1*	Rwanda	-1.506	29.615
FMNH 225206	MT149834					*Hipposideros ruber1*	Rwanda	-1.506	29.615
FMNH 225207	redundant					*Hipposideros ruber1*	Rwanda	-1.506	29.615
FMNH 225208	MT149835	MT149689	MT149587	MT149489	MT149390	*Hipposideros ruber1*	Rwanda	-1.506	29.615
FMNH 225209	redundant					*Hipposideros ruber1*	Rwanda	-1.506	29.615
FMNH 192935	MT149836	MT149690	MT149588	MT149490	MT149391	*Hipposideros ruber1*	Tanzania	-1.094	31.515
FMNH 137629	FJ347987					*Hipposideros ruber1*	Uganda	32.283	-0.005
FMNH 160358	MT149837					*Hipposideros ruber1*	Uganda	-0.989	29.614
FMNH 160359	MT149838	MT149691	MT149589	MT149491	MT149392	*Hipposideros ruber1*	Uganda	-0.989	29.614
FMNH 160361	MT149839					*Hipposideros ruber1*	Uganda	-1.041	29.580
FMNH 161040	MT149840					*Hipposideros ruber1*	Uganda	-0.245	29.819
FMNH 223866	redundant	MT149692	MT149590	MT149492	MT149393	*Hipposideros ruber1*	Uganda	-0.342	31.966
FMNH 223867	MT149841					*Hipposideros ruber1*	Uganda	-0.342	31.966
FMNH 227415	MT149842					*Hipposideros ruber2*	Central African Republic	3.033	16.410
FMNH 227416	MT149843					*Hipposideros ruber2*	Central African Republic	3.033	16.410
FMNH 227417	MT149844	MT149693	MT149591		MT149394	*Hipposideros ruber2*	Central African Republic	3.033	16.410
FMNH 149408	FJ347986	MT149694	MT149592	MT149493	MT149395	*Hipposideros ruber2*	D. R. Congo	-1.417	28.583
FMNH 149409	redundant					*Hipposideros ruber2*	D. R. Congo	-1.417	28.583
FMNH 149410	redundant	MT149695	MT149593	MT149494	MT149396	*Hipposideros ruber2*	D. R. Congo	-1.417	28.583
FMNH 149412	redundant					*Hipposideros ruber2*	D. R. Congo	-1.417	28.583
ROM 100546	FJ347978					*Hipposideros ruber2*	Ivory Coast	6.930	-7.217
FMNH 215479	redundant					*Hipposideros ruber2*	Kenya	0.244	34.907
FMNH 215480	MT149845					*Hipposideros ruber2*	Kenya	0.244	34.907
FMNH 215481	MT149846					*Hipposideros ruber2*	Kenya	0.244	34.907
FMNH 215482	redundant	MT149696	MT149594	MT149495	MT149397	*Hipposideros ruber2*	Kenya	0.244	34.907
FMNH 215483	redundant	MT149697	MT149595	MT149496	MT149398	*Hipposideros ruber2*	Kenya	0.244	34.907
NMK 184878	MT149847					*Hipposideros ruber2*	Kenya	0.248	34.906
NMK 184880	MT149848					*Hipposideros ruber2*	Kenya	0.248	34.906
NMK 184882	MT149849					*Hipposideros ruber2*	Kenya	0.248	34.906
NMK 184883	MT149850					*Hipposideros ruber2*	Kenya	0.248	34.906
NMK 184884	MT149851					*Hipposideros ruber2*	Kenya	0.248	34.906
NMK 187383	redundant					*Hipposideros ruber2*	Kenya	0.212	34.899
NMK 187384	MT149852					*Hipposideros ruber2*	Kenya	0.212	34.899
FMNH 165161	redundant					*Hipposideros ruber2*	Uganda	1.733	31.467
FMNH 165162	MT149853					*Hipposideros ruber2*	Uganda	1.683	31.533
FMNH 165163	redundant	MT149698	MT149596	MT149497	MT149399	*Hipposideros ruber2*	Uganda	1.683	31.533
FMNH 165164	MT149854					*Hipposideros ruber2*	Uganda	1.683	31.533
FMNH 165165	MT149855					*Hipposideros ruber2*	Uganda	1.683	31.533
FMNH 165166	redundant					*Hipposideros ruber2*	Uganda	1.750	31.583
FMNH 165167	redundant	MT149699	MT149597	MT149498	MT149400	*Hipposideros ruber2*	Uganda	1.733	31.467
FMNH 224069	redundant					*Hipposideros ruber2*	Uganda	0.501	30.426
FMNH 224071	redundant					*Hipposideros ruber2*	Uganda	0.501	30.426
FMNH 224074	MT149856					*Hipposideros ruber2*	Uganda	0.501	30.426
FMNH 224075	MT149857					*Hipposideros ruber2*	Uganda	0.501	30.426
	FJ347994					*Hipposideros ruber3*	Cameroon	3.564	13.408
	FJ347991					*Hipposideros ruber3*	Cameroon	2.941	9.911
	FJ347990					*Hipposideros ruber3*	Cameroon	2.941	9.911
	FJ347988					*Hipposideros ruber3*	Cameroon	4.913	9.241
EBD 18240	FJ347984					*Hipposideros ruber3*	Equatorial Guinea	1.889	9.793
EBD 18266	FJ347985					*Hipposideros ruber3*	Equatorial Guinea	1.889	9.793
EBD 18511	FJ347983					*Hipposideros ruber3*	Equatorial Guinea	3.747	8.750
EBD 18942	FJ347981					*Hipposideros ruber3*	Principe	1.615	7.404
EBD 18926	FJ347982					*Hipposideros ruber3*	São Tomé	0.219	6.727
FMNH 219477	MT149858	MT149700	MT149598	MT149499	MT149401	*Hipposideros ruber4*	D. R. Congo	-5.290	14.871
FMNH 169707	KT583815					*Macronycteris commersoni*	Madagascar	-12.932	49.057
FMNH 175777	KT583822					*Macronycteris commersoni*	Madagascar	-16.380	45.345
FMNH 175966	KT583823					*Macronycteris commersoni*	Madagascar	-22.486	45.392
FMNH 175974	MT149859					*Macronycteris commersoni*	Madagascar	-22.317	45.293
FMNH 175975	MT149860					*Macronycteris commersoni*	Madagascar	-22.317	45.293
FMNH 176155	KT583824	MT149701	MT149599	MT149500	MT149402	*Macronycteris commersoni*	Madagascar	-22.778	43.523
FMNH 176158	MT149861					*Macronycteris commersoni*	Madagascar	-22.217	43.330
FMNH 176277	redundant					*Macronycteris commersoni*	Madagascar	-12.942	49.055
FMNH 177302	KT583825					*Macronycteris commersoni*	Madagascar	-16.315	46.810
FMNH 178803	redundant					*Macronycteris commersoni*	Madagascar	-12.712	49.474
FMNH 178806	KT583816					*Macronycteris commersoni*	Madagascar	-12.712	49.474
FMNH 178808	KT583817 redundant					*Macronycteris commersoni*	Madagascar	-12.712	49.474
FMNH 178809	KT583818					*Macronycteris commersoni*	Madagascar	-12.712	49.474
FMNH 178810	KT583819					*Macronycteris commersoni*	Madagascar	-12.712	49.474
FMNH 178811	KT583820					*Macronycteris commersoni*	Madagascar	-12.712	49.474
FMNH 178812	KT583826					*Macronycteris commersoni*	Madagascar	-12.712	49.474
FMNH 178815	KT583821 redundant					*Macronycteris commersoni*	Madagascar	-12.712	49.474
FMNH 179201	MT149862					*Macronycteris commersoni*	Madagascar	-17.280	49.420
FMNH 183934	KT583827					*Macronycteris commersoni*	Madagascar	-24.050	43.750
FMNH 183980	KT583813 redundant					*Macronycteris commersoni*	Madagascar	-12.337	49.385
FMNH 184030	KT583812					*Macronycteris commersoni*	Madagascar	-14.966	47.308
FMNH 184170	KT583828					*Macronycteris commersoni*	Madagascar	-24.650	43.963
FMNH 184887	MT149863					*Macronycteris commersoni*	Madagascar	-15.904	46.598
FMNH 209236	MT149864					*Macronycteris commersoni*	Madagascar	-18.063	44.541
FMNH 217940	KT583831 redundant					*Macronycteris commersoni*	Madagascar	-22.632	45.338
FMNH 221308	KT583829					*Macronycteris commersoni*	Madagascar	-12.932	49.057
FMNH 231862	MT149865	MT149702	MT149600	MT149501	MT149315	*Macronycteris commersoni*	Madagascar	-17.889	49.203
UADBA 32916	KT583830					*Macronycteris commersoni*	Madagascar	15.538	46.886
UADBA 32987	KT583814					*Macronycteris commersoni*	Madagascar	-13.932	49.057
UADBA 32989	KR606333					*Macronycteris commersoni*	Madagascar	-12.917	49.143
FMNH 175970	MT149866	MT149703	MT149601	MT149502	MT149403	*Macronycteris cryptovalorona*	Madagascar	-22.317	45.293
FMNH 184173	MT149867	MT149704	MT149602	MT149503	MT149404	*Macronycteris cryptovalorona*	Madagascar	-24.650	43.963
AMNH 269871	KT583801					*Macronycteris gigas*	Central African Republic		
FMNH 219602	MT149868	MT149705	MT149603	MT149504	MT149405	*Macronycteris gigas*	D. R. Congo	0.241	20.883
FMNH 219682	MT149869	MT149706	MT149604	MT149415	MT149406	*Macronycteris gigas*	D. R. Congo	0.241	20.883
FMNH 220226	redundant	MT149707	MT149605	MT149505	MT149407	*Macronycteris gigas*	Kenya	-4.647	39.380
FMNH 220227	redundant	MT149708	MT149606	MT149506	MT149408	*Macronycteris gigas*	Kenya	-4.647	39.380
FMNH 220239	MT149870					*Macronycteris gigas*	Kenya	-4.215	39.451
DM 12602	KF551826					*Macronycteris gigas*	Liberia	7.553	-8.492
IVB S1032	EU934469					*Macronycteris gigas*	Senegal	12.350	-13.217
IVB S1044	EU934470					*Macronycteris gigas*	Senegal	12.350	-13.217
AMNH 269879	KT583802					*Macronycteris vittata*	Central African Republic	3.500	16.000
FMNH 215942	MT149871	MT149709	MT149607	MT149507	MT149409	*Macronycteris vittata*	Kenya	-3.300	39.995
FMNH 215943	redundant					*Macronycteris vittata*	Kenya	-3.300	39.995
FMNH 215944	redundant					*Macronycteris vittata*	Kenya	-3.300	39.995
FMNH 215945	redundant					*Macronycteris vittata*	Kenya	-3.300	39.995
FMNH 215946	MT149872					*Macronycteris vittata*	Kenya	-3.287	39.982
FMNH 215947	redundant					*Macronycteris vittata*	Kenya	-3.287	39.982
FMNH 215948	MT149873					*Macronycteris vittata*	Kenya	-3.287	39.982
FMNH 215949	redundant					*Macronycteris vittata*	Kenya	-3.287	39.982
FMNH 215950	MT149874					*Macronycteris vittata*	Kenya	-3.287	39.982
FMNH 215951	MT149875					*Macronycteris vittata*	Kenya	-3.282	39.971
FMNH 215952	MT149876					*Macronycteris vittata*	Kenya	-3.282	39.971
FMNH 215953	redundant	MT149710	MT149608		MT149410	*Macronycteris vittata*	Kenya	-3.282	39.971
FMNH 215954	MT149877					*Macronycteris vittata*	Kenya	-3.282	39.971
FMNH 215955	redundant					*Macronycteris vittata*	Kenya	-3.282	39.971
FMNH 215959	MT149878					*Macronycteris vittata*	Kenya	-3.309	40.018
FMNH 215960	MT149879					*Macronycteris vittata*	Kenya	-3.309	40.018
FMNH 215961	MT149880					*Macronycteris vittata*	Kenya	-3.309	40.018
FMNH 215962	MT149881					*Macronycteris vittata*	Kenya	-3.309	40.018
FMNH 215963	MT149882					*Macronycteris vittata*	Kenya	-3.309	40.018
FMNH 215967	MT149883					*Macronycteris vittata*	Kenya	-3.303	39.999
FMNH 215968	redundant					*Macronycteris vittata*	Kenya	-3.305	39.937
FMNH 215969	redundant					*Macronycteris vittata*	Kenya	-3.305	39.937
FMNH 220224	redundant					*Macronycteris vittata*	Kenya	-4.647	39.378
FMNH 220225	MT149885					*Macronycteris vittata*	Kenya	-4.647	39.380
FMNH 220228	MT149886					*Macronycteris vittata*	Kenya	-4.647	39.380
FMNH 220229	MT149887	MT149711	MT149609	MT149508	MT149411	*Macronycteris vittata*	Kenya	-4.647	39.380
FMNH 220231	MT149888					*Macronycteris vittata*	Kenya	-4.614	39.354
FMNH 220232	MT149889					*Macronycteris vittata*	Kenya	-4.614	39.354
FMNH 220233	MT149890					*Macronycteris vittata*	Kenya	-4.614	39.354
FMNH 220234	redundant					*Macronycteris vittata*	Kenya	-4.614	39.354
FMNH 220235	MT149891	MT149712	MT149610	MT149509	MT149412	*Macronycteris vittata*	Kenya	-4.614	39.354
FMNH 220236	redundant					*Macronycteris vittata*	Kenya	-4.614	39.354
FMNH 220237	redundant					*Macronycteris vittata*	Kenya	-4.614	39.354
FMNH 220238	redundant					*Macronycteris vittata*	Kenya	-4.614	39.354
FMNH 220240	redundant					*Macronycteris vittata*	Kenya	-3.300	39.995
NMK 187219	redundant					*Macronycteris vittata*	Kenya	-3.335	40.031
NMK 187220	MT149884					*Macronycteris vittata*	Kenya	-3.335	40.031
NMK 187221	redundant					*Macronycteris vittata*	Kenya	-3.335	40.031
NMK 187222	redundant					*Macronycteris vittata*	Kenya	-3.335	40.031
DM 8645	KF551828 redundant					*Macronycteris vittata*	Mozambique	-18.565	32.220
DM 11510	KF551827					*Macronycteris vittata*	Mozambique	-18.978	34.176
FMNH 192800	redundant	MT149713	MT149611	MT149510	MT149413	*Macronycteris vittata*	Tanzania	-4.902	39.688
FMNH 192801	MT149892					*Macronycteris vittata*	Tanzania	-4.902	39.688
FMNH 192857	redundant	MT149714	MT149612	MT149511	MT149414	*Macronycteris vittata*	Tanzania	-4.902	39.688
FMNH 192858	redundant					*Macronycteris vittata*	Tanzania	-4.902	39.688
FMNH 192859	MT149893					*Macronycteris vittata*	Tanzania	-4.902	39.688
FMNH 192860	KT583807					*Macronycteris vittata*	Tanzania	-4.902	39.688
FMNH 192865	KT583808 redundant					*Macronycteris vittata*	Tanzania	-4.902	39.688
FMNH 192866	KT583809					*Macronycteris vittata*	Tanzania	-4.902	39.688
FMNH 220268		MT149614	MT149512	MT149417	MT149316	*Triaenops afer*	Kenya	-4.590	39.331

## Supplementary materials

Supplementary material 1 Figure S1. Geographic distribution of voucher specimens used in this analysis Data type occurrence https://binary.pensoft.net/file/402781This dataset is made available under the Open Database License (http://opendatacommons.org/licenses/odbl/1.0/). The Open Database License (ODbL) is a license agreement intended to allow users to freely share, modify, and use this Dataset while maintaining this same freedom for others, provided that the original source and author(s) are credited.Bruce D. Patterson, Paul W. Webala, Tyrone H. Lavery, Bernard R. Agwanda, Steven M. Goodman, Julian C. Kerbis Peterhans, Terrence C. Demos

Supplementary material 2 Figure S2. Phylogeny of Hipposideridae based on maximum likelihood analysis of cyt-b based on 452 individuals Data type phylogenetic tree https://binary.pensoft.net/file/402782This dataset is made available under the Open Database License (http://opendatacommons.org/licenses/odbl/1.0/). The Open Database License (ODbL) is a license agreement intended to allow users to freely share, modify, and use this Dataset while maintaining this same freedom for others, provided that the original source and author(s) are credited.Bruce D. Patterson, Paul W. Webala, Tyrone H. Lavery, Bernard R. Agwanda, Steven M. Goodman, Julian C. Kerbis Peterhans, Terrence C. Demos

Supplementary material 3 Figure S3. Phylogeny of Hipposideridae based on Bayesian inference analysis of cyt-b based on 452 individuals Data type phylogenetic tree https://binary.pensoft.net/file/402783This dataset is made available under the Open Database License (http://opendatacommons.org/licenses/odbl/1.0/). The Open Database License (ODbL) is a license agreement intended to allow users to freely share, modify, and use this Dataset while maintaining this same freedom for others, provided that the original source and author(s) are credited.Bruce D. Patterson, Paul W. Webala, Tyrone H. Lavery, Bernard R. Agwanda, Steven M. Goodman, Julian C. Kerbis Peterhans, Terrence C. Demos
