# Supplementary figures and images for: Evolutionary relationships and population genetics of the Afrotropical leaf-nosed bats (Chiroptera, Hipposideridae)
Source: Zookeys. 2020 Apr 22;929:117–61. doi: 10.3897/zookeys.929.50240 (PMC7197329; doi:10.3897/zookeys.929.50240)

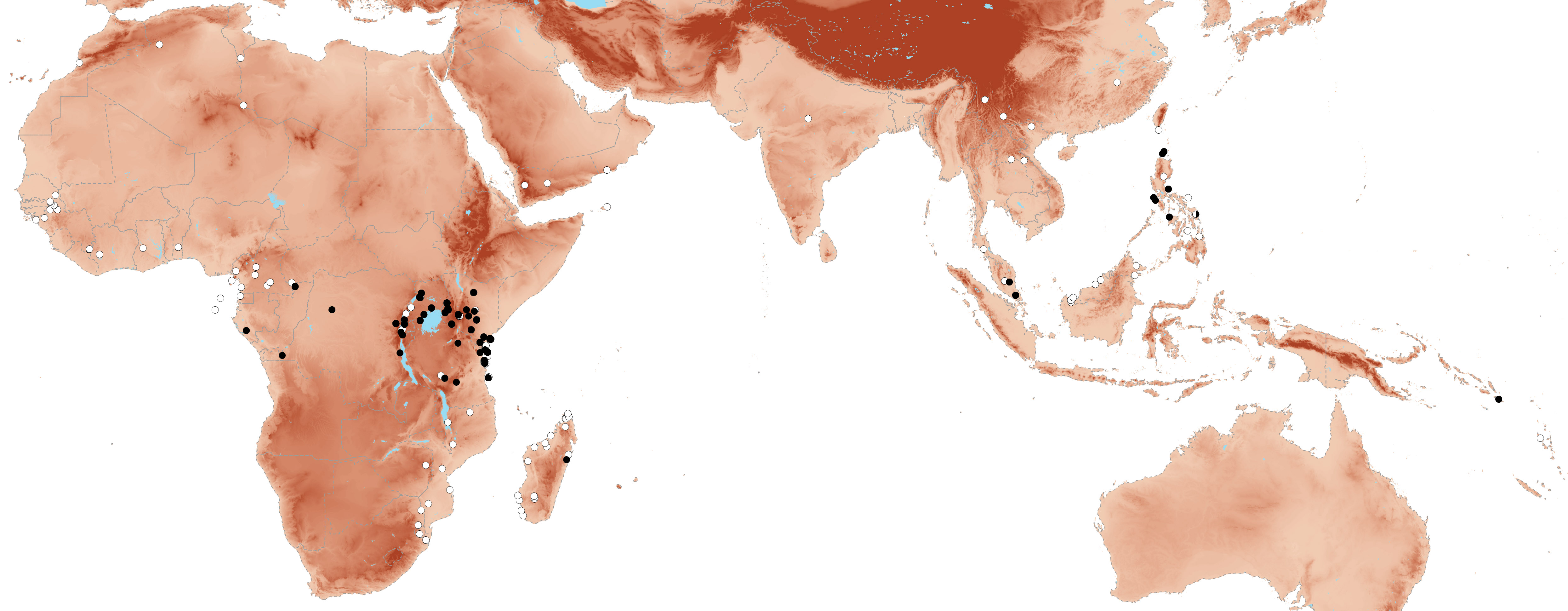

Supplement: Supplementary material 1 — Figure S1. Geographic distribution of voucher specimens used in this analysis [file zookeys-929-117-s001.jpg]

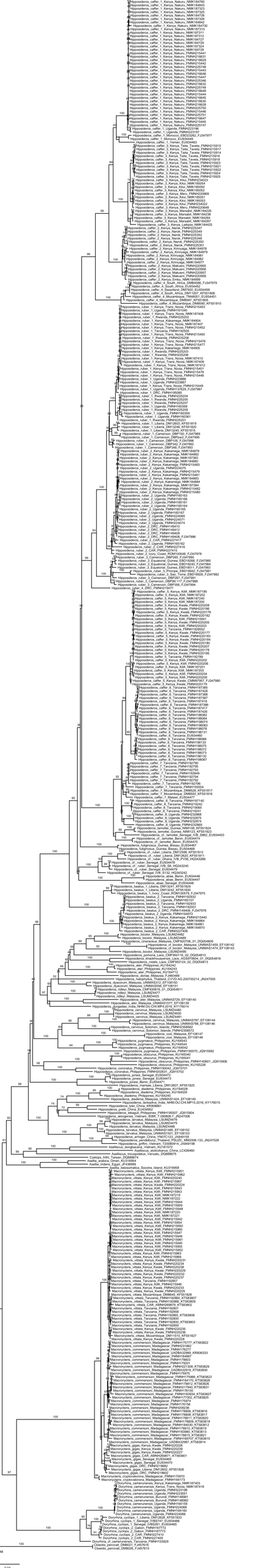

Supplement: Supplementary material 2 — Figure S2. Phylogeny of Hipposideridae based on maximum likelihood analysis of cyt-b based on 452 individuals [file zookeys-929-117-s002.pdf]

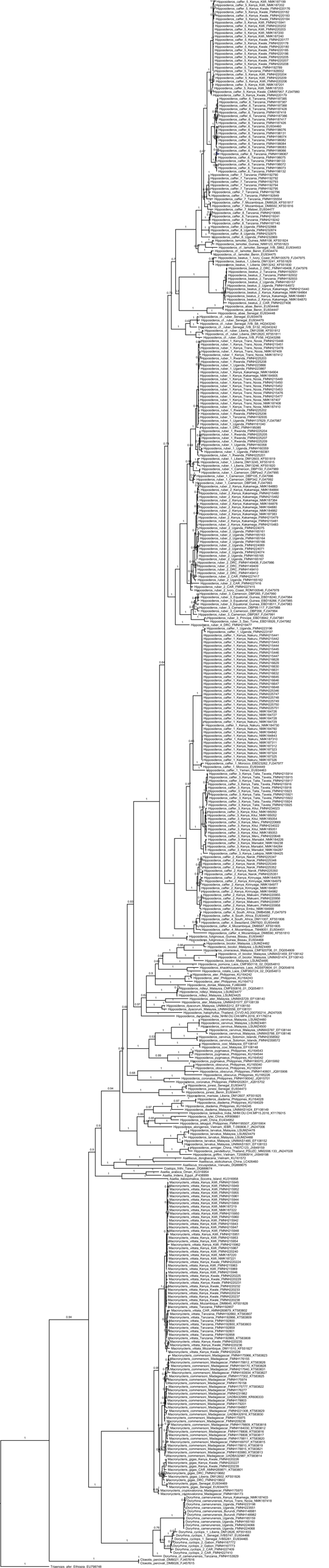

Triaenops\_ater\_Ethiopia\_EU798748

0.5

Cloecotis\_percivali\_DM8026\_FJ457615

Supplement: Supplementary material 3 — Figure S3. Phylogeny of Hipposideridae based on Bayesian inference analysis of cyt-b based on 452 individuals [file zookeys-929-117-s003.pdf]
